# Ovary Proteome Analysis Reveals RH36 Regulates Reproduction via Vitellin Uptake Mediated by HSP70 Protein in Hard Ticks

**DOI:** 10.3389/fcimb.2020.00093

**Published:** 2020-03-10

**Authors:** Fangfang Wang, Yanan Wang, Guanghua Wang, Houshuang Zhang, Ceyan Kuang, Yongzhi Zhou, Jie Cao, Jinlin Zhou

**Affiliations:** ^1^Key Laboratory of Animal Parasitology of Ministry of Agriculture, Shanghai Veterinary Research Institute, Chinese Academy of Agricultural Sciences, Shanghai, China; ^2^College of Life Science and Food Engineering, Hebei University of Engineering, Handan, China

**Keywords:** *Rhipicephalus haemaphysaloides*, RH36, vitellogenesis, RNA interference, HSP70 protein

## Abstract

Ticks are blood-sucking vector arthropods, which play an important role in transmitting pathogens between humans and animals. RH36 is an immunomodulatory protein expressed in the salivary glands, but not other organs, of partially fed *Rhipicephalus haemaphysaloides* ticks, and it reaches its peak on the day of tick engorgement. RH36 gene silencing inhibited tick blood feeding and induced a significant decrease in tick oviposition, indicating that another function of immunosuppressor RH36 was regulating tick reproduction. Why did RH36 protein expressed uniquely in the salivary gland regulate tick reproduction? RH36 regulated positively the expression of vitellogenin in ovary, which indicated RH36 protein played an important role in the integration of nutrition and reproduction. According to proteomic analysis, heat shock protein 70 (HSP70) was significantly down-regulated in the immature ovary of post-engorged ticks. In addition, gene silencing of HSP70 not only inhibited tick blood-sucking and the expression of vitellogenin, but also increased tick death rate. These results suggested RH36 affected tick vitellogenin uptake and then regulated ovary cell maturation by modulating the expression of HSP70 protein, and finally controlled tick oviposition.

## Introduction

Ticks transmit a wide range of zoonosis, such as tick-borne encephalitis (Grabowski et al., 2017), Lyme disease (Ehrmann et al., 2018), tick-borne spotted fever (Han et al., 2018), anaplasmosis (Parola and Raoult, 2001; Eisen and Eisen, 2018), and babesiosis (Kotal et al., 2015). These pathogens are transmitted via tick saliva to their vertebrate host when ticks feed on host. Transovarial transmission from adult female tick to its offspring may also occur in some tick species and tick-borne diseases (Danielova et al., 2002; Harris et al., 2017; Umemiya-Shirafuji et al., 2017; Jongejan et al., 2018; Moore et al., 2018). Tick salivary glands secrete immunosuppressant proteins including P36 protein that regulate the host immune system during blood feeding (Bergman et al., 2000; Alarcon-Chaidez et al., 2003; Konnai et al., 2009; Anatriello et al., 2010; Wang et al., 2017). Although P36 homolog proteins from Ixodid ticks are not highly conserved, all ticks share the same key site and structural features with conserved region “IDKGMLSPF”. Based on 3D structures of P36 from *Dermacentor andersoni*, a predicted conserved antigenic region was identified, located within the exposed loop with a potential of binding immunomodulating ligands, including glycerol and lactose (Oyugi et al., 2018). In our previous study, we found that RH36 from *Rhipicephalus haemaphysaloides*, P36 homolog, suppressed the T-lymphocyte mitogen-driven proliferation of splenocytes *in vitro* and *in vivo*, as well as the expression of several cytokines, including IL-2, IL-12, and TNF-α (Wang et al., 2017). P36 homologs expressed mainly in the salivary glands of partially fed ticks (Alarcon-Chaidez et al., 2003; Konnai et al., 2009) and RH36 protein production correlated with blood-feeding success and oviposition (Wang et al., 2017). Little information is known about the expression and interaction of RH36 molecules in the salivary glands and reproductive organs, including the ovary, while the mechanism by which the RH36 gene regulates ovary development is also unknown.

Ticks with a huge reproductive capacity produce hundreds of eggs, while the key of egg maturation is vitellogenesis, a process in which massive amounts of vitellin (Vn) precursors (vitellogenin, Vg) are expressed in fat bodies and midguts, and then secreted into hemolymph, and subsequently accumulated by developing oocytes via vitellogenin receptor-mediated clathrin-dependent endocytosis (Rosell and Coons, 1992; Esmeralda Parra-Peralbo, 2011; Khalil et al., 2011). After blood feeding, tick vitellogenesis is activated by a cooperative action of nutritional amino acid/target of rapamycin/S6 kinase (AA/TOR/S6K) (Umemiya-Shirafuji et al., 2012) and steroid hormone (20-hydroxyecdysone, 20E) pathways, closely correlated with mating (Thompson et al., 2005). In addition, heme and iron metabolism during tick blood feeding are essential for tick reproduction and egg fertility (Hajdusek et al., 2009; Galay et al., 2013; Perner et al., 2016). Genome and proteomics analyses of the parasitic processes seem to be unique in ticks, including prolonged feeding, new methods of hemoglobin digestion, heme detoxification, vitellogenesis and transmission of pathogens (Gulia-Nuss et al., 2016; Barrero et al., 2017). We hypothesized that the RH36 molecules participated in the integration between reproduction and nutrition and investigated the mechanism of RH36 in oviposition by proteomics analyses.

The significance of our study is revelation of the molecular mechanism of immunosuppressor RH36 modulating ovary development and oviposition in *R. haemaphysaloides* ticks. The main hypothesis addressed in this study was that RH36 may regulate tick vitellogenesis, while heat shock protein 70 (HSP70) was found to be down-regulated in the RH36 gene-silenced ticks by proteomic analysis, which regulated tick blood feeding and the expression of vitellogenin. This study illustrates the critical function of RH36 in the process of ovary development and tick reproduction, which are required for blood meal completion.

## Materials and Methods

### Ethics Statement

The protocols (shvri-ra-2017070578) were approved by the Institutional Animal Care and Use Committee of the Shanghai Veterinary Research Institute, and authorized by the Animal Ethical Committee of Shanghai Veterinary Research Institute.

### Ticks and Sample Preparation

Ticks were obtained from a colony of *R. haemaphysaloides* maintained on the ears of New Zealand white rabbits at Shanghai Veterinary Research Institute, CAAS. Off-host ticks were kept in an incubator at 25°C and 95% humidity. To detect dynamic expression of RH36 and other proteins, midguts, salivary glands, ovaries, hemolymph, and fat bodies were dissected from unfed female ticks, 5-day-fed female ticks, 7-day-fed female ticks, engorging ticks and ticks on day 3 or 10 after engorgement. Furthermore, unfed female adult ticks (*N* = 80 females per group, three replicates) were microinjected with RH36 and luciferase double-stranded RNA (dsRNA), respectively, as described previously (Wang et al., 2017). Two individual five-day fed female ticks from each group were collected to characterize RH36 mRNA levels after RH36 RNA interference (RNAi). Blood-feeding ticks from RH36 or luciferase gene silencing groups (14 ticks per group) were removed from rabbits at 5 (indicated by RF5 and LF5, respectively) or 7 days (indicated by RF7 and LF7). Engorged ticks (14 ticks per group) were also collected from RH36 or luciferase gene silencing groups at day 0 (denoted by RE0 and LE0), day 3 (denoted by RE3 and LE3), day 7 (denoted by RE7 and LE7) and day 10 (denoted by RE10 and LE10) after engorgement. The ovaries, hemolymph, fat bodies and salivary glands were dissected from 7 ticks per group for RNA or protein extraction and washed in PBS to remove hemolymph -related cells.

Total RNA was extracted from ovaries using TRIZOL (Invitrogen, Carlsbad, CA, USA) according to the manufacturer's instructions. Total RNA quality was evaluated using Agilent 2100 Bioanalyzer RNA Nano Chip (Agilent Technologies, Santa Clara, CA, USA). Tick ovaries with SDT buffer (containing 4% SDS, 100 mM DTT and 100 mM Tris-HCl, pH 7.6) and quartz sand (another 1/4 inch ceramic bead MP 6540-424 for tissue samples) were homogenized by MP homogenizer (24 × 2, 6.0M/S, 60s, twice) and subsequently sonicated and boiled for 15 min. After it was centrifuged at 10,000 rpm for 20 min, the supernatant was filtered with 0.22 μm filters. Total protein was also extracted from tick salivary glands, fat bodies and hemolymph according to the above instruction, respectively. The filtrate was quantified with the BCA Protein Assay Kit (Bio-Rad, USA). These RNA and protein samples were stored at −80°C until use.

### Antibody Against RH36 Preparation and Expression Level of RH36 Protein by Western Blot

The B cell linear epitope of RH36 protein was predicted using the Immune Epitope Database (IEDB) of http://tools.iedb.org/bcell/result/. The peptide (LTDDYWKQGEHPSGEYPIS) was selected randomly and synthesized with peptide purify of at least 95% and coupled with KLH by GL Biochem (Shanghai, China), and emulsified with an equal volume of Freund's complete adjuvant (Sigma-Aldrich, St. Louis, MO, USA) used to intraperitoneally inject Balb/c mice. Two booster injections of RH36 peptide were given with the same procedure but using Freund's incomplete adjuvant. One week after the final immunization, sera were collected following centrifugation. Antibodies against RH36 peptides were used to detect expression levels by Western blot. According to standard techniques, total proteins were separated on a 12% SDS-PAGE gel and transferred to PVDF membranes (0.45 μm, Millipore, Billerica, MA, USA), and the membranes were immunoblotted with RH36 antibody (1:200). The monoclonal antibody of β-actin against mouse (Proteintech, Rosemont, USA, Cat. No. 60008-1-Ig) was used as the control. IgG goat anti-mouse or goat anti-rabbit antibodies conjugated with HRP were used for secondary antibodies (1:8,000, Bethyl Laboratories, Inc., Montgomery, Texas, USA). The membranes were visualized using automatic chemiluminescence image analysis system (Tanon-5200, Tanon Science & Technology Co., Ltd, Shanghai, China). Densitometry used to analyze indicated protein bands was determined by Image J (NIH), normalized with the control (Schneider et al., 2012).

### Antibody Against Vitellin Preparation and Expression Level of Vitellin Protein by Western Blot

Crude eggs were homogenized and subsequently centrifuged at 10,000 rpm for 20 min, the supernatant was applied onto a Sepharose CL-4B gel filtration column and diethyl-amino-ethyl (DEAE)-cellulose column in turn (Yang et al., 2015). Polyclonal antibody and monoclonal antibody against vitellin was generated from Balb/c mice (Wang et al., 2017). Splenocytes from immunized mice were fused to myeloma cells, SP2/0, using polyethylene glycol. Hybridomas were selected in HAT medium and the hybridoma culture supernatants were screened by enzyme-linked immunosorbent assay (ELISA) in 96-microtiter plates and Western blot (Yang et al., 2015).

Expression of vitellin was detected in the ovaries, hemolymph, fat bodies and salivary glands by Western blot according to the above description. The monoclonal antibodies of β-actin (Proteintech, Cat. No. 60008-1-Ig) and GAPDH (Proteintech, Cat. No. 60004-1-Ig) against mouse were used as the control. The bands were visualized using automatic chemiluminescence image analysis system (Tanon-5200, Tanon Science & Technology Co., Ltd., Shanghai, China). Heat shock protein 70 rabbit antibody (HSP70, Cell Signaling Technology, Boston, USA, Cat. No. 3177) was also used to detect the protein expression level in the different organs during different blood-feeding ticks by Western blot. The anti-GAPDH antibody against rabbit (Cell Signaling Technology, Cat. No. 2118) was used as the control.

### Immunofluorescence Assays (IFA)

For immunofluorescence, ovaries were obtained from adult female ticks at 10 days after engorgement as described above, fixed with 4% paraformaldehyde in 0.2 M sodium cacodylate buffer, and dehydrated in a graded series of ethanol and embedded in paraffin. Sections (4 μm) were prepared and mounted on glass slides. Paraffin was removed from the sections with xylene and the sections were hydrated by successive 5-min washes with a graded series of 100, 80, 75, and 65% ethanol. The slides were treated with EDTA antigen repair buffer for 30 min at 37°C, washed with PBST and incubated with 2% bovine serum albumin (BSA; Sigma-Aldrich) in PBST for 30 min at room temperature. The slides were then incubated for 14 h at 4°C with primary antibodies against vitellin diluted 1:5–1:200 in 2% BSA mixture and developed for 30 min with goat-anti-mouse IgG conjugated with FITC (Sigma-Aldrich) (diluted 1:500 in 2% BSA mixture) after 3 washes in PBST buffer. The slides were incubated for 20 min with DAPI (1:200, 2% BSA dilution) after 3 washes in PBST buffer and mounted in Fluoromount Aqueous Mounting Medium (Sigma-Aldrich). Samples were observed and photographed with 10X objective, 1.0X zoom and the excitation of 488 nm (FITC) and 405 nm (DAPI) under a Zeiss inverted fluorescence microscope (Zeiss, Germany).

### Proteomic Data Collection and Bioinformatics Analysis

Quantitative proteomic analysis by tandem mass tag (TMT) technology was performed by Shanghai Applied Protein Technology (APT, Shanghai, China). Each 100 μg peptide mixture digested with trypsin (Promega, Madison, WI, USA) was labeled using TMT reagent (Thermo Fisher Scientific, Waltham, MA, USA) according to the manufacturer's instructions. The 6-plex TMT Labeling were fractionated into 15 strong cation exchange (SCX). LC-MS/MS analysis (1H) was performed on a Q Exactive mass spectrometer (Thermo Fisher Scientific) that was coupled to Easy nLC (Proxeon Biosystems, now Thermo Fisher Scientific) for 60 min. The mass spectrometer was operated in positive ion mode. MS data was acquired using a data-dependent top 10 method dynamically choosing the most abundant precursor ions from the survey scan (300–1,800 m/z) for HCD fragmentation. Automatic gain control (AGC) target was set to 3e6, and maximum inject time to 10 ms. Dynamic exclusion duration was 40.0 s. Survey scans were acquired at a resolution of 70,000 at m/z 200 and resolution for HCD spectra was set to 17,500 at m/z 200 and isolation width was 2 m/z. Normalized collision energy was 30 eV and the underfill ratio, which specifies the minimum percentage of the target value likely to be reached at maximum fill time, was defined as 0.1%. The instrument was run with peptide recognition mode enabled. And results were compared to a Uniprot database containing all sequences from Ixodidae (January 1, 2017) using MASCOT engine (Matrix Science, London, UK; version 2.2) embedded into Proteome Discoverer 1.4. The MASCOT identified parameters were set in Supplementary Table 1. Peptide identification was validated using false discovery rate (FDR ≤ 0.01). The standard of screening differentially expressed proteins was Fold change >1.2 and a *P*-value < 0.05 was considered statistically significant. Sequences of differentially expressed proteins were also searched using blastp tool against the online Kyoto Encyclopedia of Genes and Genomes (KEGG) database (http://geneontology.org/) and were subsequently mapped to pathways in KEGG. GO and KEGG pathway enrichment analysis were applied based on the Fisher's exact test. All assays were performed in three replicates.

### RNA Sequencing and Analysis

After total RNA was treated with DNase I, mRNA was enriched by oligo (dT) with beads and fragmented into fragments of 100–400 bp. First-strand cDNA was synthesized from the mRNA fragments. And second-strand cDNA was then synthesized using DNA polymerase I and RNaseH. Short fragments were purified, end-repaired, connected with signal nucleotide A (adenine) addition and ligated with library-specific adaptors. The cDNA library was constructed and tested in qualification by Agilent 2100 Bioanalyzer and ABI StepOnePlus Real-Time PCR System, and then sequenced using Illumina HiSeq 4000.

Tick ovary transcriptome *de novo* assembly was performed by Beijing Genomics Institute (BGI, Shenzhen, China). After sequencing, low-quality, adaptor-polluted and high content of unknown base (N) reads were filtered to get clean reads. Clean reads were assembled by Trinity assembles software to obtain high-quality contigs (Yu et al., 2015). T gicl was used to perform clustering and eliminate redundant data in the assembled transcripts to obtain unique genes. Subsequently, expression analysis, heterozygous SNP detection, and functional annotation were performed. Differentially expression genes (DEGs) were further analyzed with gene ontology functional enrichment and KEGG pathway functional enrichment. The false discovery rate (FDR ≤ 0.001) was used to determine the threshold *P* value in multiple tests. Results were the mean of three replicates.

### HSP70 RNA Interference (RNAi) for Gene Knockdown in Ticks

The dsRNA was synthesized using the T7 RiboMAX™ RNAi System (Promega, Madison, WI, USA) according to the manufacturer's recommendations. Oligonucleotide primers containing T7 promoter sequences (in italics at 5′-end) were listed in Supplementary Table 2. A dsRNA targeting luciferase used as a negative control was subjected to the same PCR amplification protocol using luciferase-specific primers (Yu et al., 2013). These dsRNA were maintained at −80°C until use. Unfed adult ticks (*N* = 50 females per group, two independent groups) were microinjected with 0.5 μL HSP70 dsRNA (about 1 μg RNA) at the base of the fourth right leg of the ventral surface of ticks. Control ticks were microinjected with unrelated luciferase dsRNA. After microinjection, ticks were maintained in an incubator at 25°C and 95% humidity for 24 h and then allowed to feed on rabbits' ears. Four female ticks per group were collected at 5 days of blood feeding for RNA and protein extraction to characterize gene knockdown by real-time quantitative PCR and Western blot with respect to luciferase control. Remaining ticks were allowed to feed until engorgement, which tick mortality, engorgement rate and engorgement weight was determined in individual engorged female ticks.

### Determination of Tick mRNA Levels by Real-time Quantitative PCR

The primers and amplicon length of target genes used for real-time quantitative PCR are listed in Supplementary Table 3. The first-stranded cDNA was synthesized from 1 μg RNAs with the PrimeScript RT reagent kit (Perfect Real Time) (Takara, Shiga, Japan) in the following program: 37°C for 15 min, 85°C for 7 s, and finally 4°C. Quantitative real-time PCR was performed using SYBR Premix Ex Taq (Takara, Japan) with QuantStudio 5 (ABI, Waltham, USA) in 20 μL reaction mixtures including 0.4 μL ROX Dye II. The reactions were incubated for 30 s at 95°C, followed by 40 cycles at 95°C for 5 s, 60°C for 34 s, and analysis of a melting curve. Tick elongation factor 1α (ELF1α) was used as an internal control because of its stability in *R. microplus* and *R. appendiculatus* ticks (Nijhof et al., 2009). All reactions were performed in triplicate. The 2^−Δ*Ct*^ method was used to calculate relative expression levels.

### Statistical Analysis

The body weights of engorged ticks were expressed as the mean ± standard error of mean (SEM) and analyzed using Student *t*-test. Differences in the attachment rate, engorgement rate, and death rate of ticks in different groups were tested using the Chi-square test. Significance of quantitative real-time PCR results was expressed as means ± SEM and determined with the one-way analysis of variance (ANOVA), and statistical analysis was conducted using software SPSS version 20.0. *P* < 0.05 and 0.01 were considered to be statistically significant (^*^) and extremely significant (^**^), respectively.

## Results

### The Immunomodulatory Protein RH36 Regulated the Complex Changes of Vitellin

**Figure 1:**
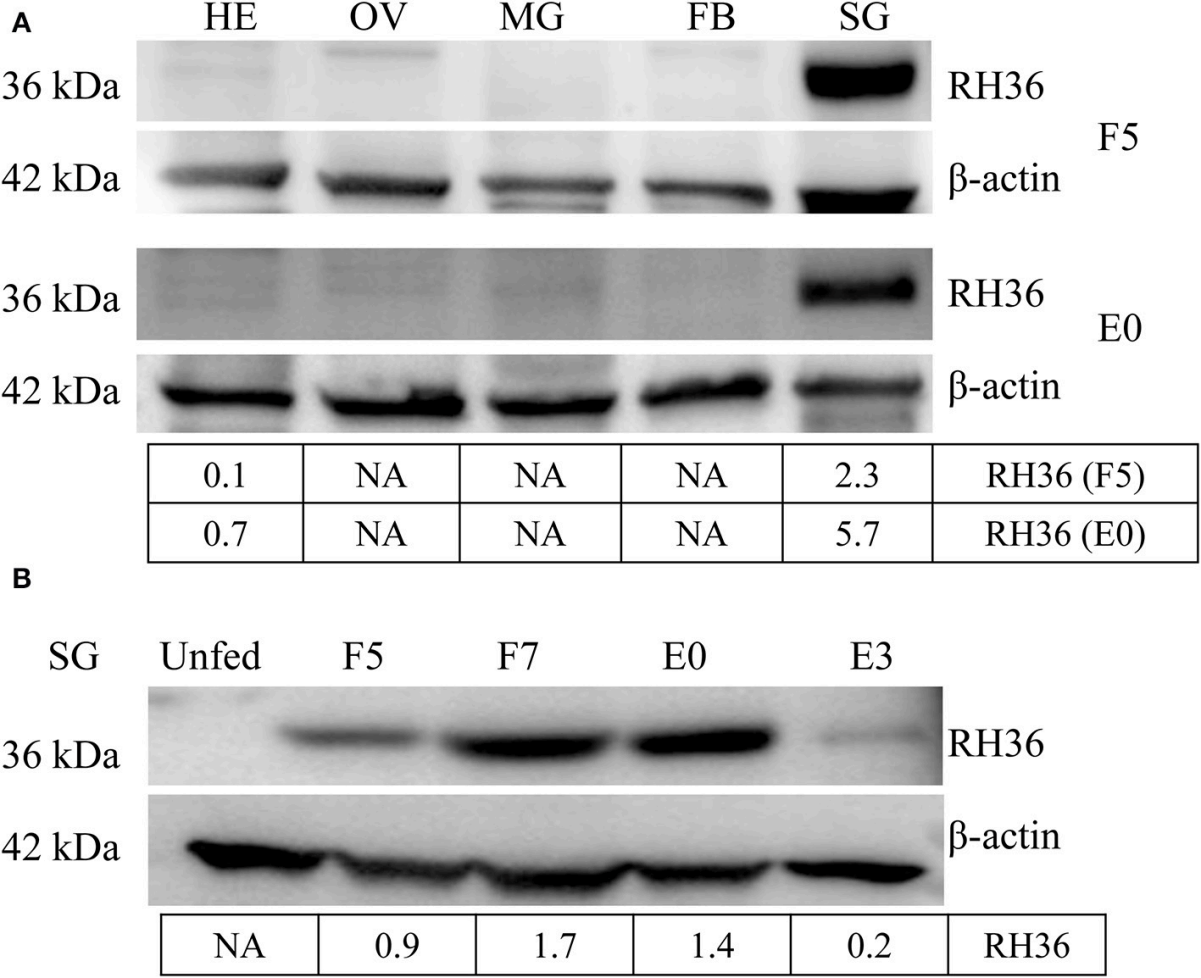
RH36 is expressed uniquely in the salivary gland and expressed higher in 7-day-fed ticks and engorged ticks. Expression level of RH36 was detected at different organs from 5-day-fed ticks (upper panel, **A**) and engorged ticks (lower panel, **A**), and different development stages **(B)** using polyclonal antibody prepared by KLH binding RH36 polypeptide. Density analysis shows RH36 protein is induced by feeding and expressed characteristically in salivary glands. The protein level of RH36 was normalized to β-actin. FB, fat body; HE, hemolymph; OV, ovary; SG, salivary gland; MG, midgut; F5, at day 5 for blood feeding; F7, at day 7 for blood feeding; E0, on the day of engorgement; E3, at 3 days post-engorgement.

RH36 protein was expressed exclusively in the salivary glands, not in the midguts, ovaries or fat bodies of 5-day-fed ticks and engorged ticks (Figure 1A). Additionally, RH36 protein was expressed at higher levels in 7-day-fed and saturated ticks and subsequently decreased after engorgement, which was highly consistent with the time of tick ovarian development (Figure 1B). To confirm our hypothesis that RH36 modulating tick ovary development via vitellogenesis, four vitellogenin genes (named RHVg1, RHVg2, RHVg3, and RHVg4, respectively) were cloned from ovarian transcriptome from the post-engorgement *R. haemaphysaloides* female ticks (Supplementary Figure 1). These sequences are highly homologous in *Haemaphysalis longicornis* and *Dermacentor variabilis* (Supplementary Figure 2). RHVg1, RHVg2, RHVg3, RHVg4 sequences were available in the GenBank databases under the accession numbers: MK584634, MK584635, MK584636, MK584637, respectively. In addition, RHVg1 (Supplementary Figure 3A), RHVg2 (Supplementary Figure 3B) and RHVg3 (Supplementary Figure 3C) genes were widely distributed in the different development stages of ticks and RHVg4 (Supplementary Figure 3D) was detected only in engorged adults not larvae and nymphs by real-time quantitative PCR and semi-quantitative PCR (Supplementary Figure 3E). What's more, RHVg2 and RHVg4 was expressed in the fat body and ovary of engorged adult ticks, respectively (Supplementary Figure 3F).

**Figure 2:**
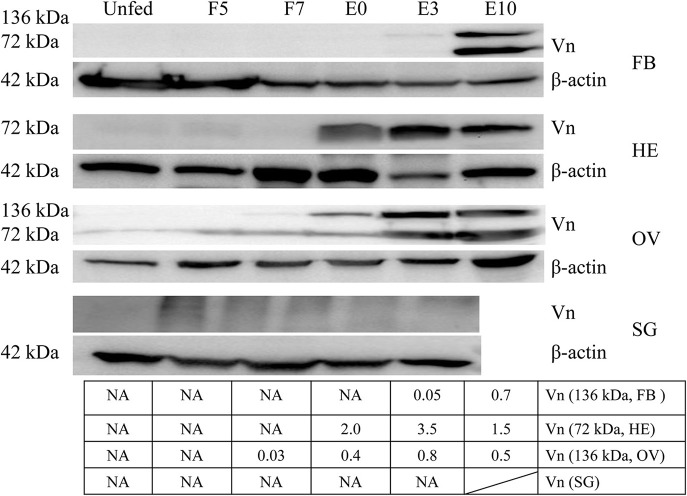
Vitellin expression is induced by blood feeding. Except for salivary gland, vitellin protein was expressed highly in fat body, hemolymph, ovary at different engorgement statuses by Western blot. The intensities of vitellin protein bands (136 and 72 kDa) were determined, normalized to β-actin. Vn, Vitellin; FB, fat body; HE, hemolymph; OV, ovary; SG, salivary gland; UF, unfed; F5, at day 5 for blood feeding; F7, at day 7 for blood feeding; E0, on the day of engorgement; E3, at 3 days post-engorgement; E10, at day 10 after engorgement.

Vitellin was purified from crude egg extraction by gel filtration (Sepharose CL-4B) and ion exchange chromatography on DEAE-cellulose and identified by possible polypeptide bands with molecular weights of 43.0, 47.3, 64.4, 72.6, 79, 85.9, 94, 102.3, 108.7, 136.5, and 161.5 kDa, respectively (Supplementary Figure 4A), in which two polypeptides (136.5, 72.7 kDa) were identified by LC-MS/MS (Supplementary Tables 4, 5). Monoclonal antibodies were generated and had specific immunological reaction with two polypeptides (136.5, 72.7 kDa) of vitellin (Supplementary Figure 4B). Vitellin proteins were highly expressed in fat body, hemolymph and ovary of adult ticks after engorgement (Figure 2).

**Figure 3:**
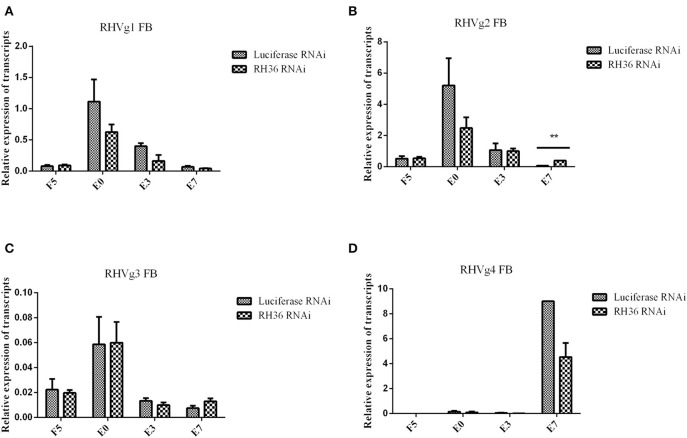
Expression of RHVg mRNA in fat body of female ticks is not affected after RH36 gene silencing. Transcript levels of RHVg1 **(A)**, RHVg2 **(B)**, RHVg3 **(C)**, and RHVg4 **(D)** were detected in the fat body of 5-day-fed ticks, engorgement ticks, 3 day post-engorgement ticks and 7 day post-engorged ticks by real-time quantitative PCR. The data were normalized to tick elongation factor 1α and gene-specific standards were the respective plasmids. RHVg2 mRNA was up-regulated significantly at 7 day post-engorgement (*p* < 0.01), but there was no difference for other RHVg genes. ** means highly statistical significance that *p*-values less than 0.01. FB, fat body; F5, at day 5 for blood feeding; E0, on the day of engorgement; E3, at 3 days post-engorgement; E7, at 7 days after engorgement.

**Figure 4:**
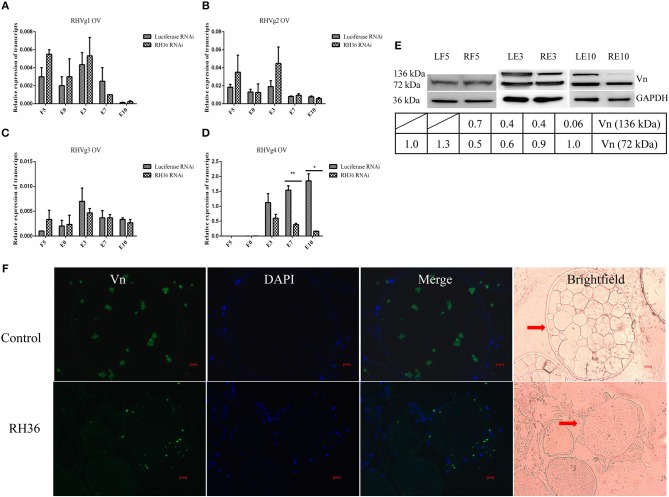
RH36 is involved in expression and/or processing of RHVg genes and vitellin protein after engorgement. Transcript level of RHVg1 **(A)**, RHVg2 **(B)**, RHVg3 **(C)**, RHVg4 **(D)** genes was tested in the ovaries of adult ticks at 5 days blood feeding, engorgement, 3 days post-engorgement, 7 days post-engorgement and 10 days post-engorgement by real-time quantitative PCR, which shows RHVg4 gene is observably down-regulated at 7 days post-engorgement (*p* < 0.01) and 10 days post-engorgement (*p* < 0.05). ** means highly statistical significance that P-values less than 0.01. * means statistical significance that *p*-values less than 0.05. The data were normalized to tick elongation factor 1α and gene-specific standards were the respective plasmids. Protein level of vitellin reduced in the ovaries at 3 days post-engorgement and 10 days post-engorgement by Western blot **(E)**. The intensities of vitellin protein bands (136 and 72 kDa) were normalized to GAPDH. RH36 regulates tick ovary development by reducing vitellin uptake in oocytes using immunofluorescence assay **(F)**. Tick ovaries were collected at 10 days after engorgement from RH36 and luciferase (as control) RNAi groups and stained with mouse anti-vitellin antibodies. Vitellin was observed in green fluorescent and DNA was stained with DAPI (blue fluorescent). Scale bar: 20 μm. LF5, 5-day-fed ticks from luciferase RNAi groups; RF5, 5-day-fed ticks from RH36 RNAi groups; E0, on the day of engorgement; LE3, 3 days post-engorgement ticks from luciferase RNAi groups; RE3, 3 days post-engorgement ticks from RH36 RNAi groups; E7, at day 7 after engorgement; LE10, 10 days post-engorgement ticks from luciferase RNAi groups; RE10, 10 days post-engorgement ticks from RH36 RNAi groups; Vn, Vitellin; OV, ovary, arrows point to oocytes.

After RH36 gene silencing, there was no difference for four vitellogenin genes in the fat body of adult ticks (Figures 3A,C,D). There was one exception: RHVg2 gene was up-regulated significantly at 7 days after engorgement (*p* < 0.01, Figure 3B). In the ovaries, although there was no difference in the transcript level of RHVg1 (Figure 4A), RHVg2 (Figure 4B) and RHVg3 (Figure 4C) genes from blood feeding to post-engorgement, RHVg4 gene was down-regulated significantly at 7 days or 10 days post engorgement (*p* < 0.01, Figure 4D). In addition, vitellin (the band of 136 kDa) protein decreased in the ovary of adult ticks at 3 or 10 days after engorgement, but not at 5 days of blood feeding (Figure 4E). Compared to mature oocytes from luciferase gene-silenced adult ticks, immunofluorescence showed that vitellin protein was less expressed in the cytoplasm of immature oocytes in response to RH36 gene-silencing (Figure 4F).

### The Importance of HSP70 in the Ovary Development in Response to RH36 Depletion

**Figure 5:**
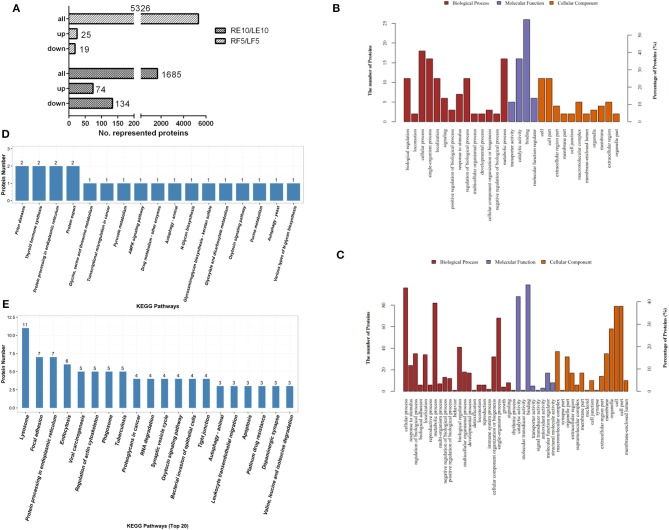
Vitellogenin and HSP70 were expressed differentially in the ovary proteomic analyses. Twelve tick ovary samples were collected from RH36 or luciferase gene-silenced adult ticks at 5 days blood feeding and 10 days post-engorgement and analyzed using TMT technology. Number of total proteins and differentially-expressed proteins were obtained from ovary samples of adult ticks **(A)**. Differentially expressed proteins mainly involved in cellular process, developmental process and metabolic process with function of lipid transporter activity, catalytic activity and binding in the 5-day-fed tick ovaries **(B)** and 10-day post-engorged tick ovaries **(C)** by GO analysis, in which vitellogenin and HSP70 involved in protein export and protein processing in endoplasmic reticulum using KEGG enrichment analysis, respectively **(D)**. Differentially expressed proteins in the 10-day post-engorged tick ovaries chiefly participate in focal adhesion, endocytosis and protein processing in endoplasmic reticulum using KEGG enrichment analysis **(E)**.

To identify which proteins regulate ovary development, ovaries from adult female ticks at 5 days blood feeding and 10 days post-engorgement were selected to conduct TMT proteomics analysis. Because of significant difference in total proteins found in ovaries between blood feeding and post-engorgement (Supplementary Figure 5), the TMT Mass-Tagging mass spectrometric (2, 6-plex) results were analyzed using MASCOT engine version 2.2. Ovary proteomics analysis resulted in the identification of 5,326 and 1,685 unique proteins in ticks at 5 days feeding and 10 days post-engorgement respectively. In the 5-day-fed ovaries, there were 44 proteins statistically significant (*p* < 0.05 and fold change > 1.2). Among these proteins, 25 and 19 proteins were up- and down-regulated, respectively (Figure 5A and Supplementary Table 6). In the day 10 post-engorgement ovaries, RH36 gene knockdown resulted in 208 proteins with statistically significant production differentiation (*p* < 0.05 and fold change > 1.2). Among these proteins, 74 were up-regulated and 134 were down-regulated (Figure 5A and Supplementary Table 7). According to GO and KEGG analyses, differentially expressed proteins mainly correlated with cellular process, developmental process and metabolic process with function of lipid transporter activity, catalytic activity and binding in the ovaries at 5 days feeding (Figure 5B) and 10 days post-engorgement (Figure 5C), in which vitellogenin (Supplementary Figure 6A) and HSP70 (Supplementary Figure 6B) involved in protein export and protein processing in endoplasmic reticulum, respectively (Figures 5D,E).

### RH36 Gene Regulated the Expression of HSP70 Protein

**Figure 6:**
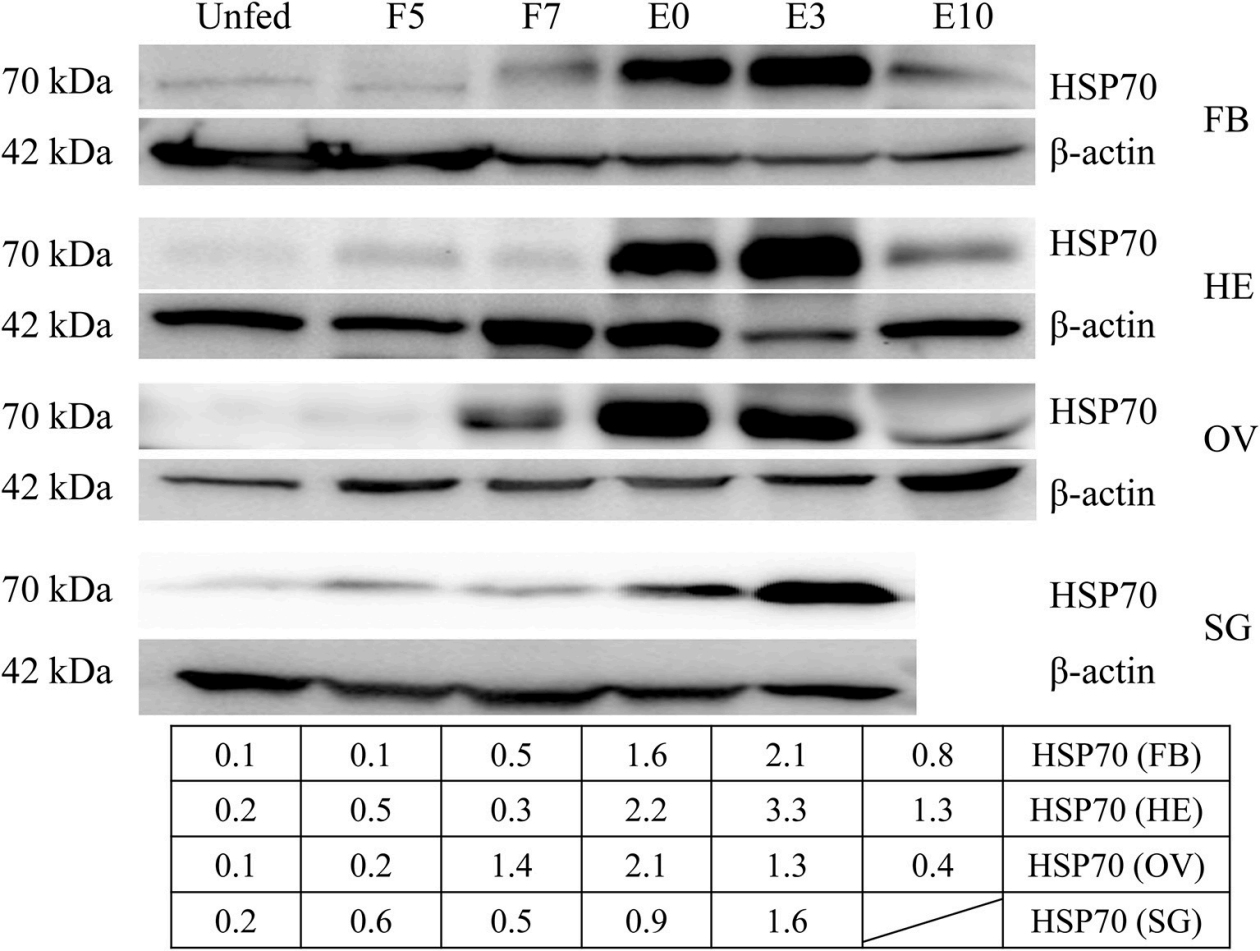
HSP70 protein was widely distributed in a variety of organs and accelerated by blood feeding. HSP70 protein was expressed widely in fat body, hemolymph, ovary and salivary gland of adult ticks at 5 days blood feeding, 7 days blood feeding, engorgement, 3 days post-engorgement and 10 days post-engorgement by Western blot. The intensities of HSP70 protein were determined and normalized to β-actin. β-actin bands were used in the previous Figure 2. FB, fat body; HE, hemolymph; OV, ovary; SG, salivary gland; UF, unfed; F5, at day 5 for blood feeding; F7, at day 7 for blood feeding; E0, on the day of engorgement; E3, at 3 days post-engorgement; E10, at day 10 after engorgement.

Since HSP70 protein was very important in tick blood-sucking and ovary maturation, we obtained HSP70 gene sequence (GenBank accession numbers: MK584638) from the ovary transcriptome (Table 1) and found that the open reading frame (ORF) of HSP70 was 1,980 bp long, encoding a deduced protein with 660 amino acids in length, a predicted molecular mass of 72.5 kDa and a pI of 5.32. It was also found that HSP70 protein included three signature sequences and the extreme C-terminal endoplasmic reticulum targeting sequence (KDEL, Supplementary Figure 7A). As shown in Supplementary Figure 7B, HSP70 from *R. haemaphysaloides* was highly conserved with over 83% identity in arthropods and mammals, in which it was highly homologous in *Haemaphysalis flava* and *Ixodes scapularis* with the identity of 96.21 and 91.06%, respectively. HSP70 protein was widely distributed in the hemolymph, ovary, salivary gland and fat body of post-engorgement ticks, with highest levels in the ovary at 7 days blood feeding and in the circulating hemolymph at 3 days post-engorgement (Figure 6).

**Table 1:** List of heat shock 70 kDa protein in the tick ovaries transcriptome.

**Unigenes**	**Subject_id**	**Gene Name**	**Species**	**Identity**	**Align length**	**Miss_match**	**E_value**	**Score**
Unigene10768_All	ACA84007.1	Heat shock 70 kDa protein 5	*Haemaphysalis longicornis*	100	21	0	1.01E-11	45.4394
CL2077.Contig1_All	ACA84007.1	Heat shock 70 kDa protein 5	*Haemaphysalis longicornis*	97.51	401	10	0	761.14
Unigene11678_All	XP_013782730.1	Heat shock 70 kDa protein 4-like	*Limulus polyphemus*	53.91	831	383	0	869.766
Unigene13971_All	P19120.2	Heat shock 70 kDa protein 8	*Bos taurus*	93.68	174	11	3.24E-86	321.627
Unigene16158_All	XP_012264348.1	Heat shock 70 kDa protein cognate 5	*Athalia rosae*	79.97	694	139	0	1061.21
Unigene18598_All	XP_001012263.1	Heat shock 70 kDa protein	*Tetrahymena thermophila*	76	75	18	6.00E-24	114.39
Unigene19775_All	XP_007453496.1	Heat shock 70 kDa protein	*Lipotes vexillifer*	61.64	219	84	3.08E-68	263.462
Unigene23136_All	XP_013788178.1	Heat shock 70 kDa protein 12A-like	*Limulus polyphemus*	72.4	616	170	0	920.613
Unigene48580_All	AIS39468.1	Heat shock 70 kDa protein	*Haemaphysalis flava*	100	20	0	2.31E-10	45.4394
Unigene48580_All	DAA34432.1	Heat shock 70 kDa protein	*Amblyomma variegatum*	95	20	1	5.35E-10	41.5874
Unigene56143_All	XP_010125825.1	Heat shock 70 kDa protein	*Chlamydotis macqueenii*	88.61	79	9	3.87E-34	148.288
CL434.Contig1_All	XP_014239635.1	Major heat shock 70 kDa protein Ba-like	*Cimex lectularius*	78.74	635	135	0	1012.29
CL2742.Contig1_All	XP_013778668.1	Heat shock 70 kDa protein cognate 4	*Limulus polyphemus*	89.68	649	67	0	1162.52

**Figure 7:**
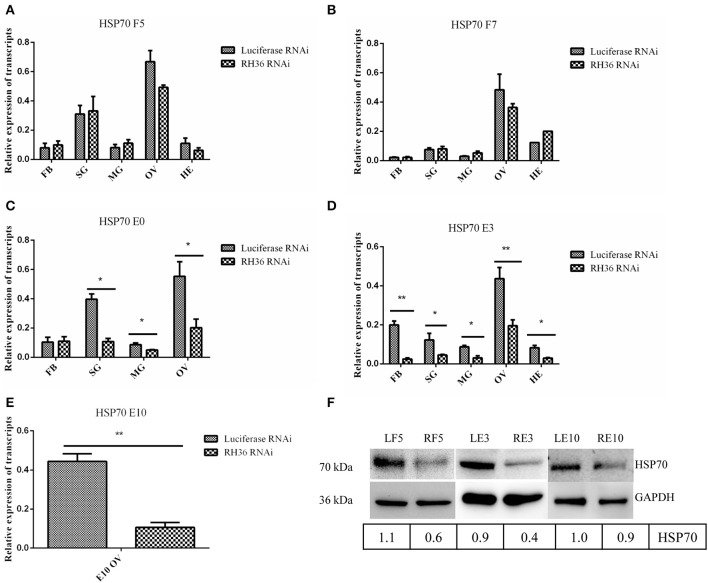
RH36 gene silencing inhibits expression of HSP70 protein after engorgement. Transcript level of HSP70 was detected in the fat body, salivary gland, midgut, ovaries and hemolymph at 5 days blood feeding **(A)**, 7 days blood feeding **(B)**, engorgement **(C)**, 3 days post-engorgement **(D)**, and 10 days post-engorgement **(E)** by real-time quantitative PCR, results show HSP70 gene is observably down-regulated in the different organs at engorgement, 3 days post-engorgement and 10 days post-engorgement (*p* < 0.05 or *p* < 0.01). The data were normalized to tick elongation factor 1α and gene-specific standard was the HSP70 plasmid including open reading frame. ** means highly statistical significance that P-values less than 0.01. * means statistical significance that *p*-values less than 0.05. Protein level of HSP70 reduced in the ovaries at 5 days blood feeding, 3 days post-engorgement and 10 days post-engorgement by Western blot **(F)**. The intensities of HSP70 were normalized to GAPDH. GAPDH bands appeared in the previous Figure 4. GAPDH bands were re-used due to LF5, 5-day-fed ticks from luciferase RNAi groups; RF5, 5-day-fed ticks from RH36 RNAi groups; F7, at day 7 for blood-feeding; E0, on the day of engorgement; LE3, 3 days post-engorgement ticks from luciferase RNAi groups; RE3, 3 days post-engorgement ticks from RH36 RNAi groups; LE10, 10 days post-engorgement ticks from luciferase RNAi groups; RE10, 10 days post-engorgement ticks from RH36 RNAi groups; FB, fat body; HE, hemolymph; OV, ovary; MG, midgut; SG, salivary gland.

After RH36 gene knocking down, there was no difference for HSP70 gene expression in the fat body, salivary gland, midgut, ovary and hemolymph of 5-day-fed and 7-day-fed adult ticks (Figures 7A,B, respectively). On the day of engorgement, HSP70 gene was down-regulated significantly in the ovary, midgut and salivary gland of adult ticks (*p* < 0.05, Figure 7C), coinciding with RH36 down-regulation expression. At 3 days post-engorgement, HSP70 gene also decreased markedly in the fat body, ovary, midgut, salivary gland, and hemolymph of adult ticks (*p* < 0.05, Figure 7D). In addition, HSP70 gene expression reduced observably in the ovary of adult ticks at 10 days post-engorgement (*p* < 0.01, Figure 7E). From tick blood-sucking to engorgement, HSP70 protein level decreased in the ovaries of adult ticks from RH36 RNAi groups by Western blot (Figure 7F). These results suggested that RH36 promoted expression of HSP70 protein in the post-engorged adult ticks.

### Impacts of HSP70 Gene Knockdown on Tick Blood Feeding

**Figure 8:**
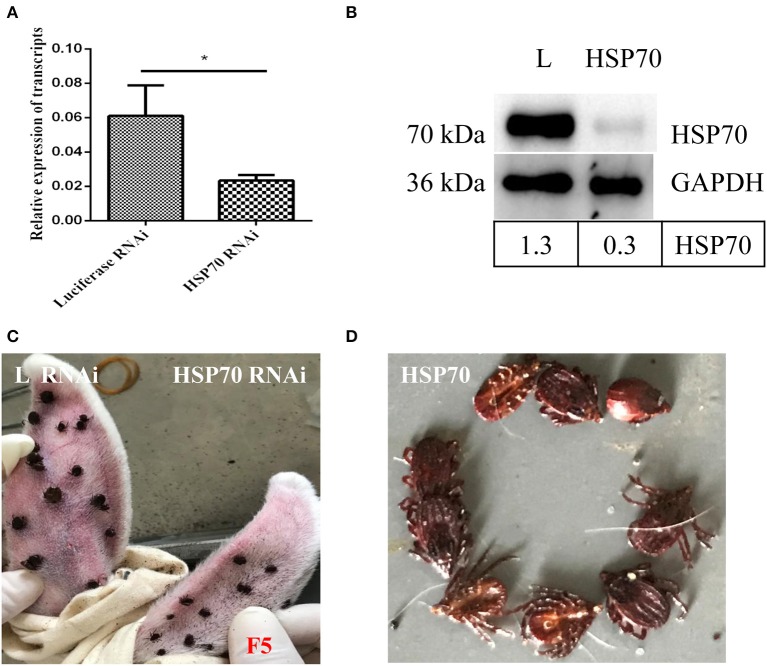
Effect of HSP70 gene knockdown on tick blood feeding. Relative expression level of HSP70 was determined by real-time quantitative PCR **(A)** and Western blot **(B)** after interference with HSP70 dsRNA vs. luciferase dsRNA. The data were normalized to tick elongation factor 1α and gene-specific standard was the HSP70 plasmid. * means statistical significance that *p*-values less than 0.05. Tick attachment was observed at 5 days blood feeding **(C,D)**. It shows HSP70 gene-knocked down inhibits blood feeding and increases tick death. F5, 5-day-fed ticks; L, luciferase RNAi groups.

**Figure 9:**
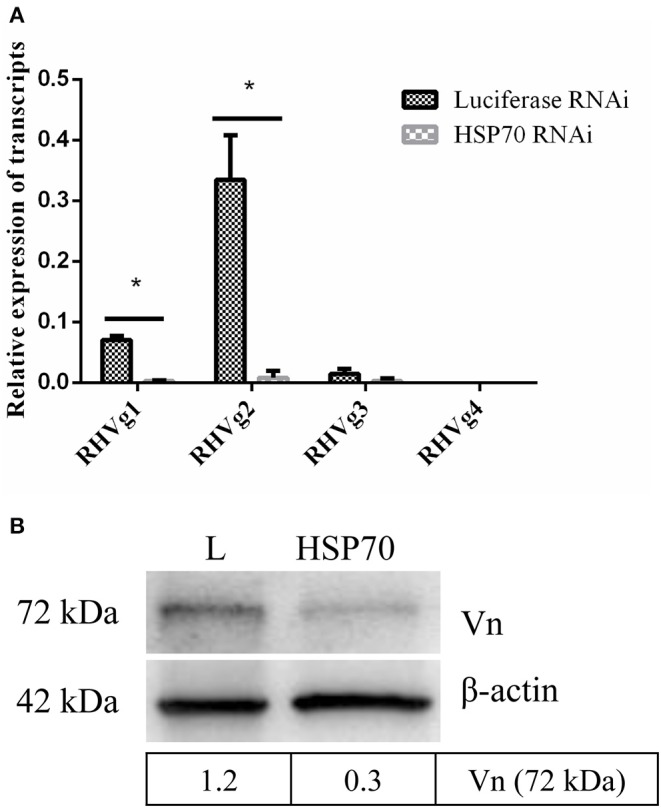
HSP70 gene silencing down regulates expression of Vg and vitellin. RHVg1 and RHVg2 genes decreased significantly in 5-day-fed ticks by real-time quantitative PCR (**A**, *p* < 0.05). ELFIa gene served as the endogenous control. Every group has three biological repeats and each repeat is three times. Data are calculated with 2^−Δ*CT*^. Gene-specific standards were the respective plasmid of RHVg genes. Meanwhile, vitellin protein was down-regulated dramatically at 5 days blood feeding using Western blot (**B**, *p* < 0.05). The intensities of vitellin protein bands (72 kDa) were determined, normalizing to β-actin. * means statistical significance that *p*-values less than 0.05. L, luciferase RNA interference, Vn, Vitellin.

HSP70 mRNA decreased by 73.86% (Figure 8A) and HSP70 protein was significantly down-regulated after microinjecting HSP70 dsRNA (*p* < 0.05, Figure 8B). After HSP70 gene silencing, mean of 48 h attachment rate and average engorgement rate was 52.22 and 2.56%, respectively. Meanwhile, these parameters showed 88.89 and 80.39% in the luciferase RNAi groups, respectively, which revealed that down-regulation of HSP70 gene decreased tick blood feeding and engorgement rate (Table 2). Although ticks attached to rabbit ears after HSP70 gene silencing (Figure 8C), the engorged body weight of ticks decreased and death rate increased compared to the luciferase RNAi groups (*p* < 0.05, Table 2 and Figure 8D), which shows that HSP70 gene facilitates abundance of tick blood feeding and protects ticks from death. After HSP70 gene knockdown, two vitellogenins (RHVg1 and RHVg2, Figure 9A) and vitellin (Figure 9B) were observably down-regulated (*p* < 0.05), indicating that HSP70 protein regulated expression of vitellogenin proteins via promoting tick blood feeding.

**Table 2:** Effect of HSP70 gene knockdown on tick blood feeding and death rate.

**Groups**	**48 h attachment rate (%)^a^**	**Engorgement rate (%)^a^**	**Engorged body weight (mg)^b^**	**Death rate (%)^a^**
HSP70	52.22 (47/90)	2.56 (2/78)	208.95 ± 201.53 (2 ticks)	97.44 (76/78)
L	88.89 (80/90)	80.39 (41/51)	324.45 ± 85.24	19.61 (10/51)

a *Significantly different using the chi-square test*.

b *Significantly different using Student's t-test*.

### RH36 Gene Played an Important Role in the Integration of Nutrition Uptake and Reproduction

**Figure 10:**
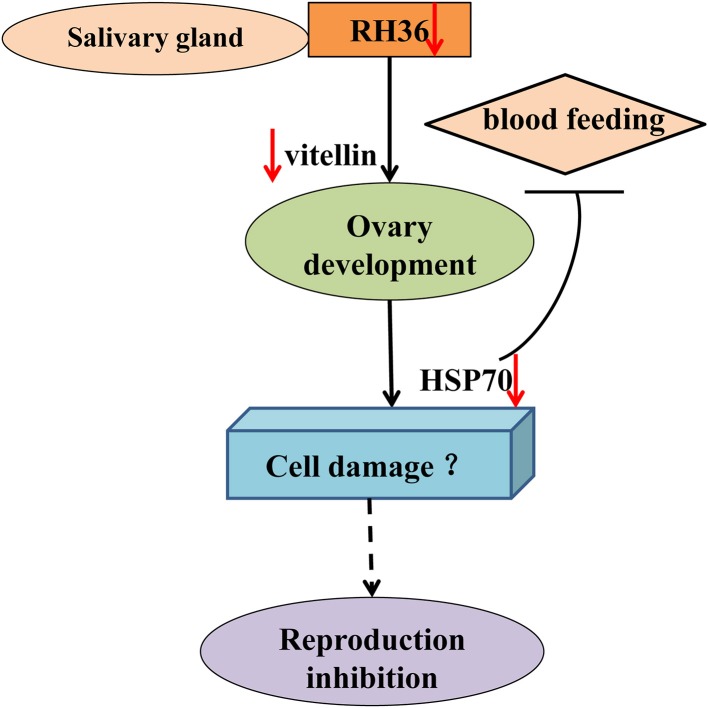
Model of RH36 regulated tick oviposition via nutrition uptake. RH36 expressed-uniquely in salivary gland regulates tick blood feeding and ovary development by inhibiting vitellin uptake mediated by HSP70 protein using real-time quantitative PCR and Western blot. In addition, HSP70 gene silencing considerably increased tick death rate during blood feeding but it is still to be further studied that RH36 gene regulates tick oviposition by accelerating cell damage via HSP70.

As shown in Figure 10, RH36 gene expressed-uniquely in salivary gland controlled tick blood feeding and modulated tick ovary development and oviposition by regulating the expression of vitellin in ovary, while HSP70 protein was down-regulated significantly in the ovaries of post-engorged ticks after RH36 gene silencing by real-time quantitative PCR and Western blot (*p* < 0.05). HSP70 gene positively modulated tick blood feeding and ovary development via nutrition uptake. Therefore, we speculated that RH36 gene silencing hindered tick blood feeding and oocytes maturity by inhibiting vitellin uptake mediated by HSP70 protein. In addition, HSP70 gene silencing considerably increased tick death rate during blood feeding.

## Discussion

RH36 not only is an immunomodulatory protein that suppressed the proliferation of splenocytes and expression of several cytokines such as IL-2, IL-12, and TNF-α, but also modulates blood feeding and oviposition in hard ticks (Wang et al., 2017). Moreover, RH36 was induced by blood feeding and expressed specifically in salivary glands, but not in other organs such as ovary and hemolymph (Figure 1). How did RH36 regulate tick ovary development indirectly? We speculated that RH36 was involved in the integration of nutrition and reproduction in ticks, in which vitellogenin is critical to tick ovary maturation since it serves as the nutritional source for tick survivability and reproduction (Xavier et al., 2018). Vitellogenin and vitellin are structurally, biochemically and immunologically similar in the majority of insects, including ticks, which results in McAb against vitellin recognized vitellogenins (Kaufman, 2004; Yang et al., 2015). Four vitellogenin genes (Supplementary Figure 1) and McAb against vitellin (Figure 4) were obtained to test the dynamics of vitellogenesis. Vitellogenin genes were widely distributed in fat body, midgut, hemolymph and ovaries of fed and engorged adult ticks (Figure 2 and Supplementary Figure 3). It is reported that vitellogenin is synthesized in the fat body and midgut of female ticks after mating and transported through the hemolymph, captured by surface receptors called vitellogenin receptors (VgRs), and endocytosed into developing oocytes within the ovaries (Rosell and Coons, 1992; Khalil et al., 2011; Mitchell et al., 2019). RH36 gene did not regulate transcripts of RHVg1, RHVg3, and RHVg4 genes in the fat body of adult ticks (Figures 3A,C,D), but RHVg2 gene in fat body was up-regulated at 7 days post-engorgement (Figure 3B), which may be stimulated by down-regulation of vitellin protein in the ovaries of 7-day-engorged ticks (Figure 4D). RHVg4 gene and vitellin protein decreased significantly in the post-engorged ovaries after RH36 gene silencing (*p* < 0.05, Figures 4A–E), and vitellin protein was less expressed in the cytoplasm of immature oocytes compared to mature oocytes at 10 days after engorgement (Figure 4F), which suggested RH36 gene silencing attenuated vitellin expression in ovaries at post-engorgement. Due to the absence of available genome for *Rhipicephalus haemaphysaloides* ticks, some ovarian-specific proteins may be lost by searching Ixodidae database resulting in less proteins correlating with reproductive process in proteomic analysis (Figure 5C). Differentially-expressed proteins relating to endocytosis played an important role in vitellogenesis (Figures 5C,E). It is suggested that RH36 may modulate vitellin uptake via endocytosis. Clathrin heavy chain involved in endocytic pathway regulates expression of vitellogenin in *R. haemaphysaloides* (Kuang et al., 2020). Vitellin is a hemoglycolipoprotein consisting of many bands (James and Oliver, 1997; Yang et al., 2015). Similarly, there were 11 polypeptides of vitellin identified by proteomic analysis in the *R. haemaphysaloides* ticks and vitellin antibody in this study recognized two bands of 136 and 72 kDa (Supplementary Figure 4) that is different from the peptides of vitellogenin in ovary proteomics analysis (Supplementary Tables 4, 5), resulting in inconsistent phenomenon.

The ovarian transcriptome from post-engorged *R. haemaphysaloides* females was analyzed and revealed that the transcripts related to lipid metabolism and other metabolisms were differentially expressed. These transcripts may play an important role in ovary development after tick engorgement. The metabolism processes could be involved in the increase of fat body cells observing in the vitellogenesis/oogenesis processes (Denardi et al., 2009). In addition, the abundance of metabolic transcripts could be related to changes in the reproductive tissues during the oogenesis/vitellogenesis processes, such as ovary size growth and oocyte maturation (Moreira et al., 2017), which is consistent with the result that RH36 gene silencing affects tick ovary development and oocytes maturity (Wang et al., 2017).

In the ovary proteomic analysis of ticks fed for 5 days, HSP70 was a typical stress protein in response to micro-injection of RH36 dsRNA (Villar et al., 2010). In the 10 days post-engorged ovary, HSP70 was inhibited significantly after RH36 gene silencing (*p* < 0.05, Figures 5A, 7) and related closely to protein processing in endoplasmic reticulum (Figure 5E). Recent work has shown that heat shock proteins are secreted to the cell exterior partially in response to stress, we hypothesize that RH36 may modulate ovary development indirectly via mediator HSP70 protein. On the one hand, HSP-70 like protein in salivary gland of ticks participates in variable fibrinogenolysis and accelerates engorged quantity upon blood feeding (Vora et al., 2017), immunomodulatory protein RH36 may be dependent on HSP70 protein to modulate tick blood feeding and nutrient supply (Wang et al., 2017). On the other hand, HSP70, as a endoplasmic reticulum chaperone, exhibits both pro-inflammatory and anti-inflammatory properties, depending on the context in which they encounter responding immune cells (Pockley and Henderson, 2018). HSP70 may have a synergistic effect with RH36 protein in salivary gland of adult ticks. However, the mechanism of HSP70 gene regulating tick oocytes development was still to be further identified. Furthermore, it is still unknown that RH36 gene regulates tick oviposition by accelerating cell death via HSP70.

After HSP70 gene silencing, the mean of 48 h attachment rate, average engorgement rate and expression of vitellin significantly decreased (*p* < 0.05, Table 2 and Figure 9). This indicates that the down-regulation of HSP70 gene decreases tick blood feeding and nutrition uptake. In ticks, blood feeding triggered salivary gland degeneration (L'Amoreaux et al., 2003) and ovary maturation (Friesen and Kaufman, 2009), suggesting vitellin protein reduced by HSP70 gene silencing inhibits tick ovary development. Starvation activates cell apoptosis and increases tick death. Consistent with this, HSP70 gene silencing dramatically increased tick death rate (Table 2 and Figure 8). We speculated that nutrition deficiency accelerated HSP70-mediated cell damage in ticks (Figure 10). The expression of HSP with antioxidant activity extends lifespan via regulating the downstream protein autophagy-related gene 7 (Morrow et al., 2004; Liao et al., 2008; Chen et al., 2012; Sarup et al., 2014; Vos et al., 2016), while HSP depletion reduces the number of viviparous offspring and simultaneously increases the number of premature nymphs, suggesting an unexpected role in aphid embryogenesis and eclosion (Will et al., 2017). Therefore, it is suggested that RH36 gene regulates vitellogenin uptake via HSP70 protein and extends lifespan of ovary to improve oviposition rate.

## Data Availability Statement

The Transcriptome Shotgun Assembly project has been deposited at DDBJ/EMBL/GenBank under the accession GIJA00000000.

## Ethics Statement

The animal study was reviewed and approved by the Institutional Animal Care and Use Committee of the Shanghai Veterinary Research Institute, and authorized by the Animal Ethical Committee of Shanghai Veterinary Research Institute.

## Author Contributions

FW planned the experiments and wrote the paper. FW, YW, GW, and CK performed the experiments. FW, HZ, and JZ analyzed the data. JC and YZ contributed to the reagents or ticks.

### Conflict of Interest

The authors declare that the research was conducted in the absence of any commercial or financial relationships that could be construed as a potential conflict of interest.
