# Supplementary material for: Ovary Proteome Analysis Reveals RH36 Regulates Reproduction via Vitellin Uptake Mediated by HSP70 Protein in Hard Ticks
Source: Front Cell Infect Microbiol. 2020 Mar 10;10:93. doi: 10.3389/fcimb.2020.00093 (PMC7076983; doi:10.3389/fcimb.2020.00093)
Supplement: Supplementary file 1 [file Data_Sheet_1.zip › Frontiers_Supplementary_Material/Supplementary_Material.docx]

Supplementary Material

# Supplementary Data

Supplementary Material includes some supplementary tables (table S1, table S4-S7) and original pictures for immunobloting.

# Supplementary Figures and Tables

## Supplementary Figures


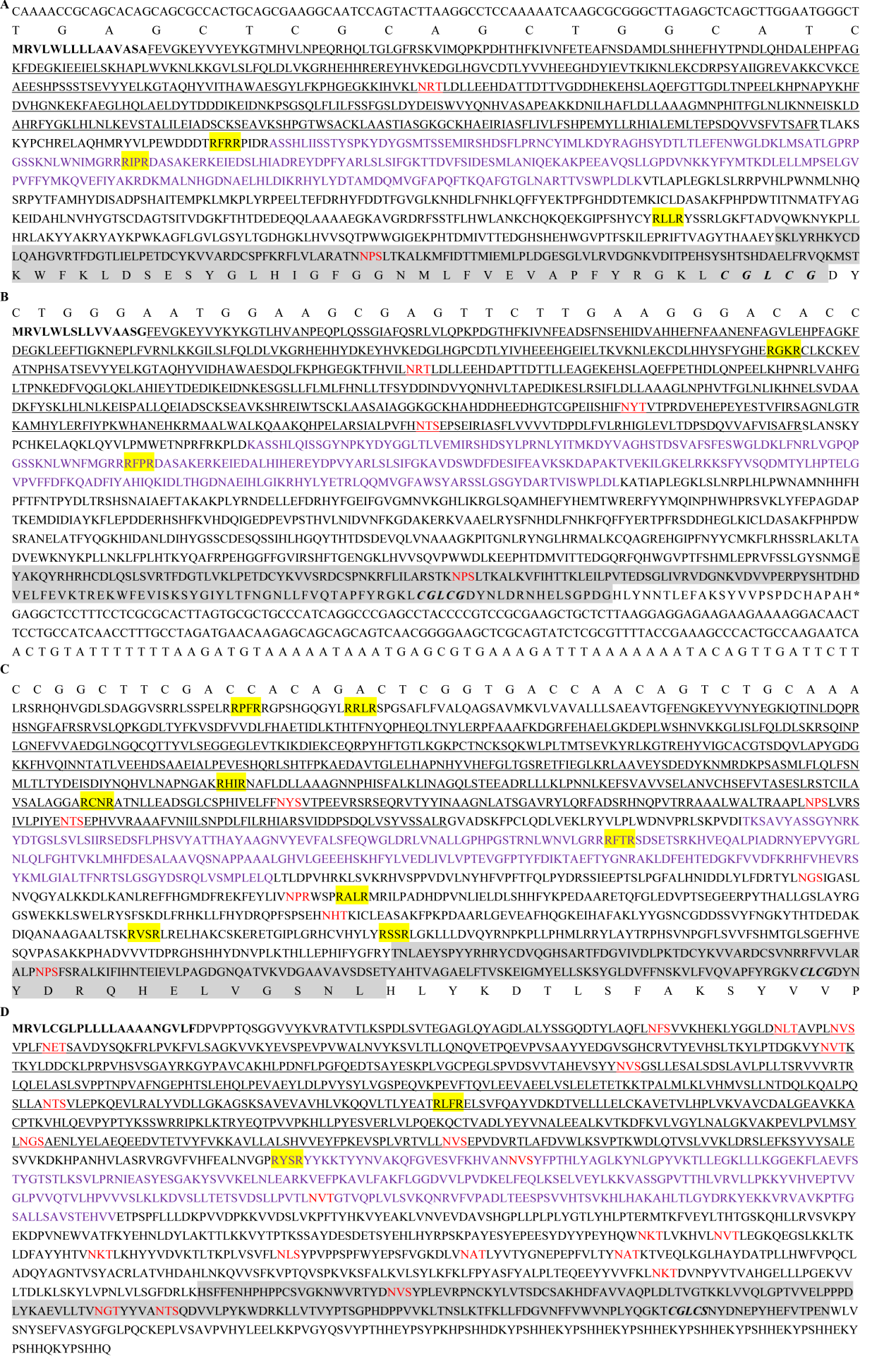


**Supplementary Figure 1.** **Primary structure of the RHVg deduced amino acid sequences.** (A) RHVg1 (GenBank accession no. MK584634); (B) RHVg2 (GenBank accession no. MK584635); (C) RHVg3 (GenBank accession no. MK584636); (D) RHVg4 (GenBank accession no. MK584637). For each RHVg, the putative signal peptide is indicated by boldface letters. Consensus RXXR sequences for possible cleavage sites are enclosed in yellow shading and the CGLCG motif, followed by cysteine residues, is shown with italic boldface letters. N-glycosylation sites are shown with red letters. The N-terminal lipoprotein domain is underlined, the carboxy von Willebrand factor type D domain is shown in blue shading, and the DUF1943 domain is marked with purple letters. The ORF stop codon is marked with an asterisk.


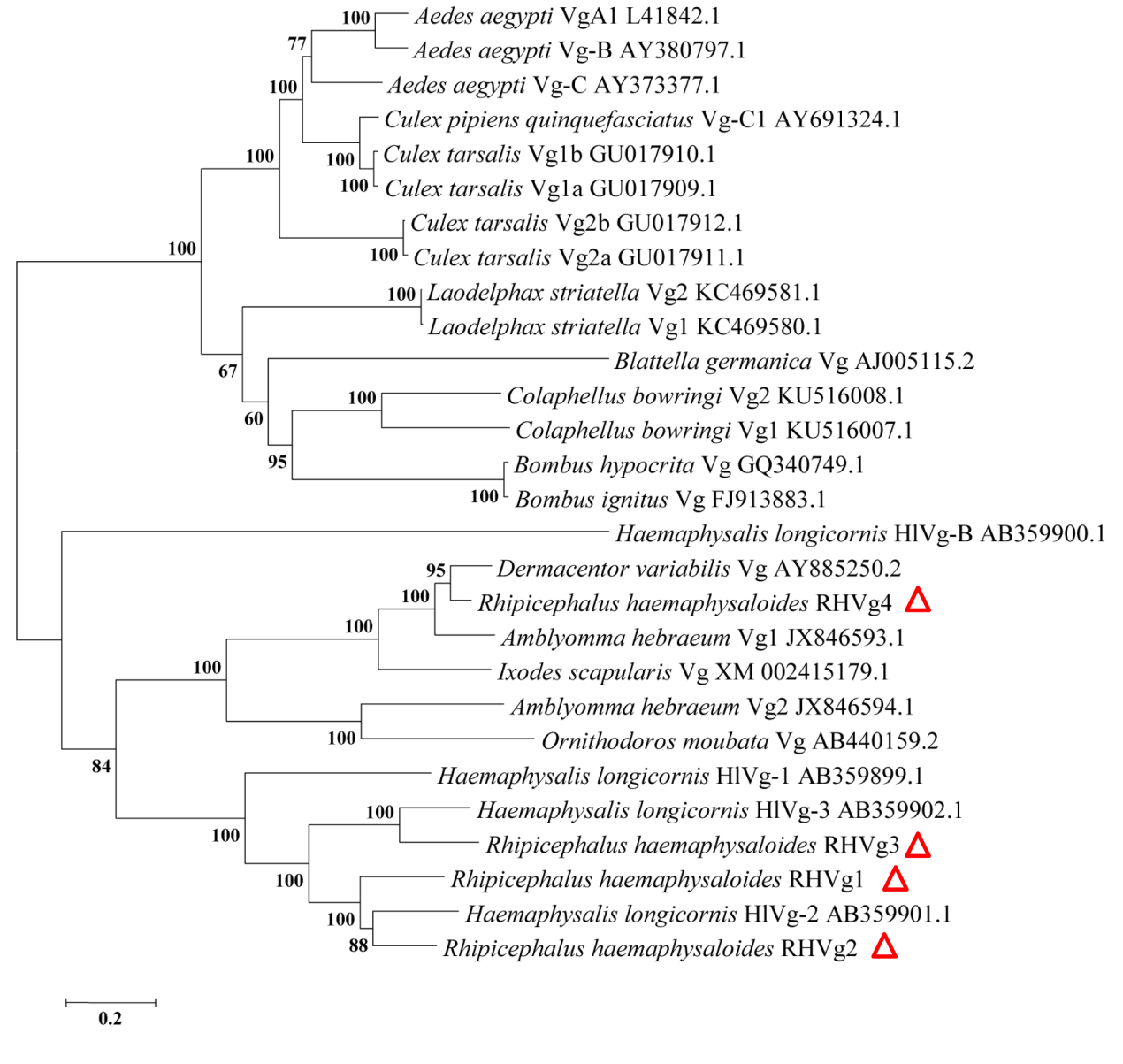


**Supplementary Figure 2. Phylogenetic tree of RHVg from various ticks and insects.** A phylogenetic tree of RHVg genes from representative species with the accession numbers was constructed by software MEGA 5.0 with Neighbor-joining tree and 100 bootstrap replications based on available amino acid sequences of typical species. △ means RHVg genes from *R. haemaphysaloides*.


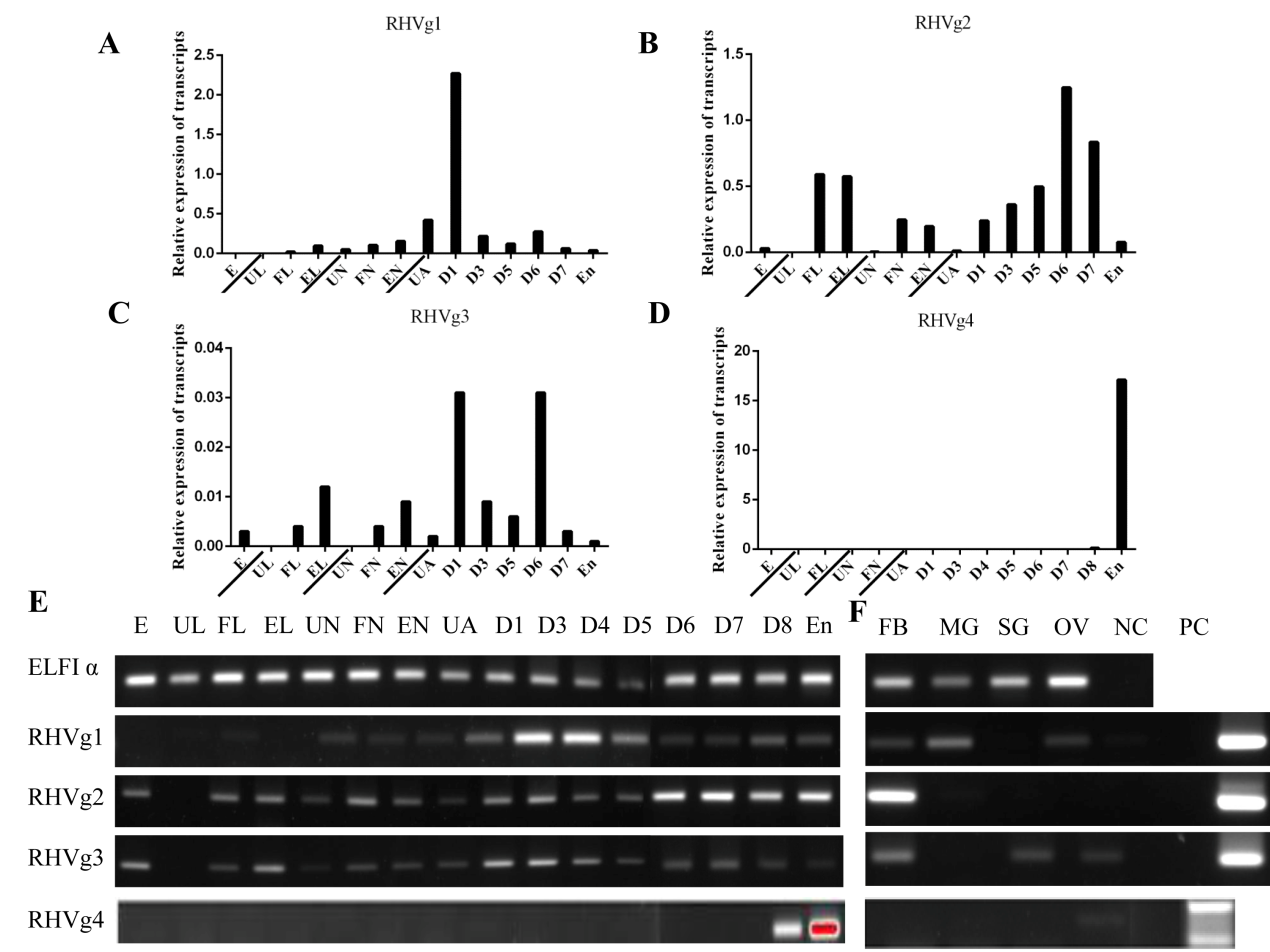
**Supplementary Figure 3. Relative expression of RHVg mRNA at different development stages during blood feeding period and different organs of fed ticks.** Transcript level of RHVg1 (A), RHVg2 (B), RHVg3 (C) and RHVg4 (D) genes at the different development stages during blood feeding period by real-time quantitative PCR; (E) Transcript level of four Vg genes at the different development stages during blood feeding period by semi-quantitative PCR; (F) Transcript level of four Vg genes at different organs from fed adult ticks by semi-quantitative PCR. E: eggs; UL: unfed larvae; FL: fed larvae; EL: engorged larvae; UN: unfed nymphs; FN: fed nymphs; EN: engorged nymphs; UA: unfed adult ticks; D1: one-day-fed ticks; D3: three-day-fed ticks; D4: four-day-fed ticks; D5: five-day-fed ticks; D6: six-day-fed ticks; D7: seven-day-fed ticks; D8: eight-day-fed ticks; En: engorged tick. RT-PCR was performed on cDNA from organs of partially fed adult females (fed day 6). FB: fat body; MG: midgut; SG: salivary glands; OV: ovary; NC: negative control; PC: positive control.

**
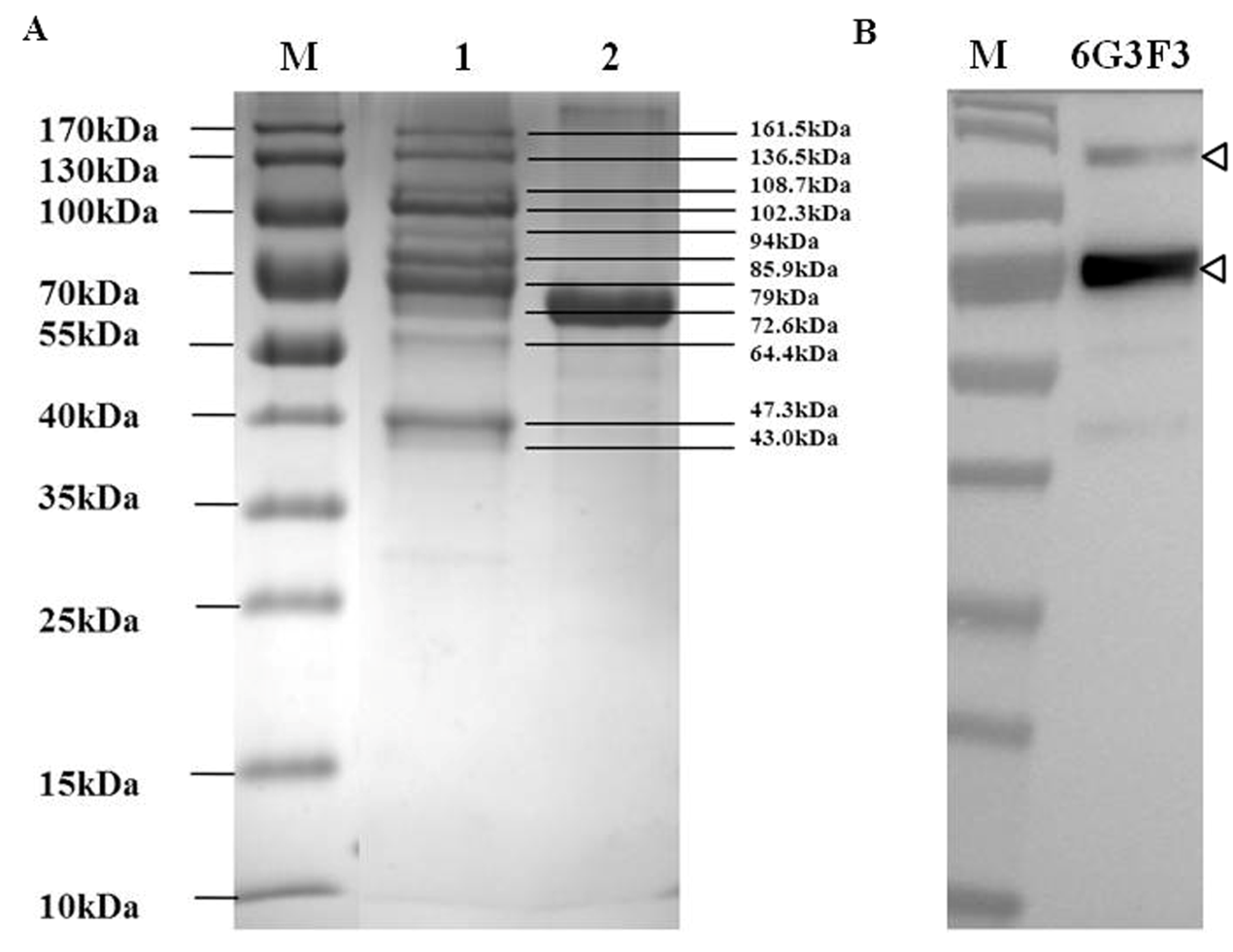
**

**Supplementary Figure 4. Analysis of purified vitellin protein.** (A) Polypeptide of vitellin separated by 10% SDS-PAGE (stained with Coomassie blue). M means marker, line 1 represents vitellin, line 2 represents BSA protein. (B) Vitellin monoclonal antibody (clone 6G3F3) identified by Western blot.


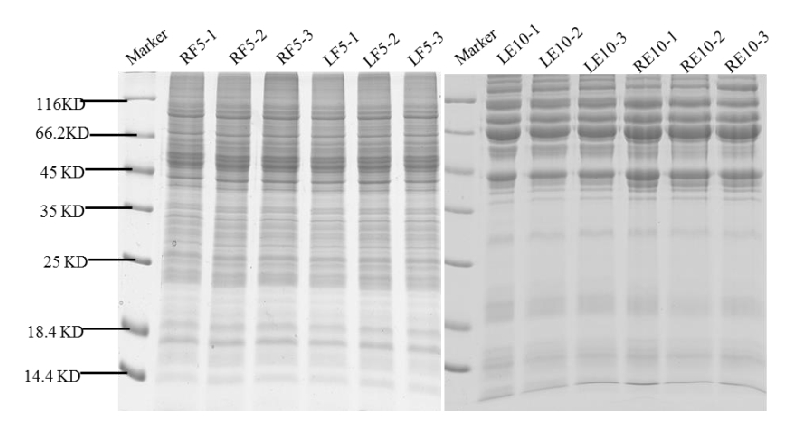


**Supplementary Figure 5.** **Total proteins SDS-PAGE of tick ovary samples.** Ovaries were collected from adult ticks at 5 days fed and 10 days post-engorgement and extracted for total proteins. Bands of total proteins were observed by SDS-PAGE (triplicate). Proteins in the ovaries of 5-day-fed ticks were various while proteins in the ovaries of 10-day-engorged ticks contained mainly macromolecular protein. LF5: 5-day-fed ticks from luciferase RNAi groups, RF5: 5-day-fed ticks from RH36 RNAi groups, LE10: 10 days post-engorgement ticks from luciferase RNAi groups, RE10: 10 days post-engorgement ticks from RH36 RNAi groups.

**
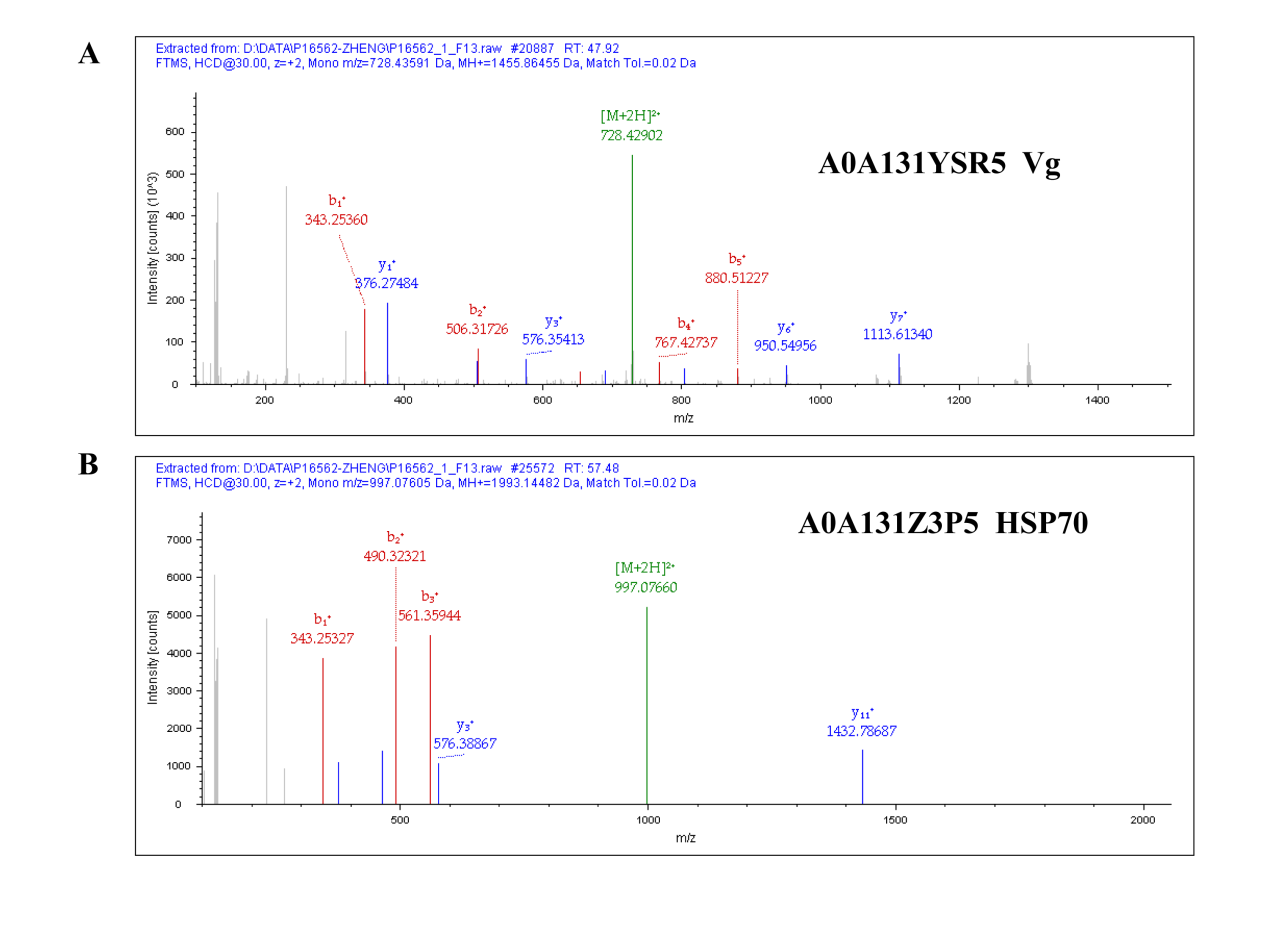
**

**Supplementary Figure 6. Representative MS/MS spectra of secondary peptide of target proteins.** The secondary spectrum diagram of Vg (A) and HSP70 (B) peptides is shown with the secondary b, y ion.

**
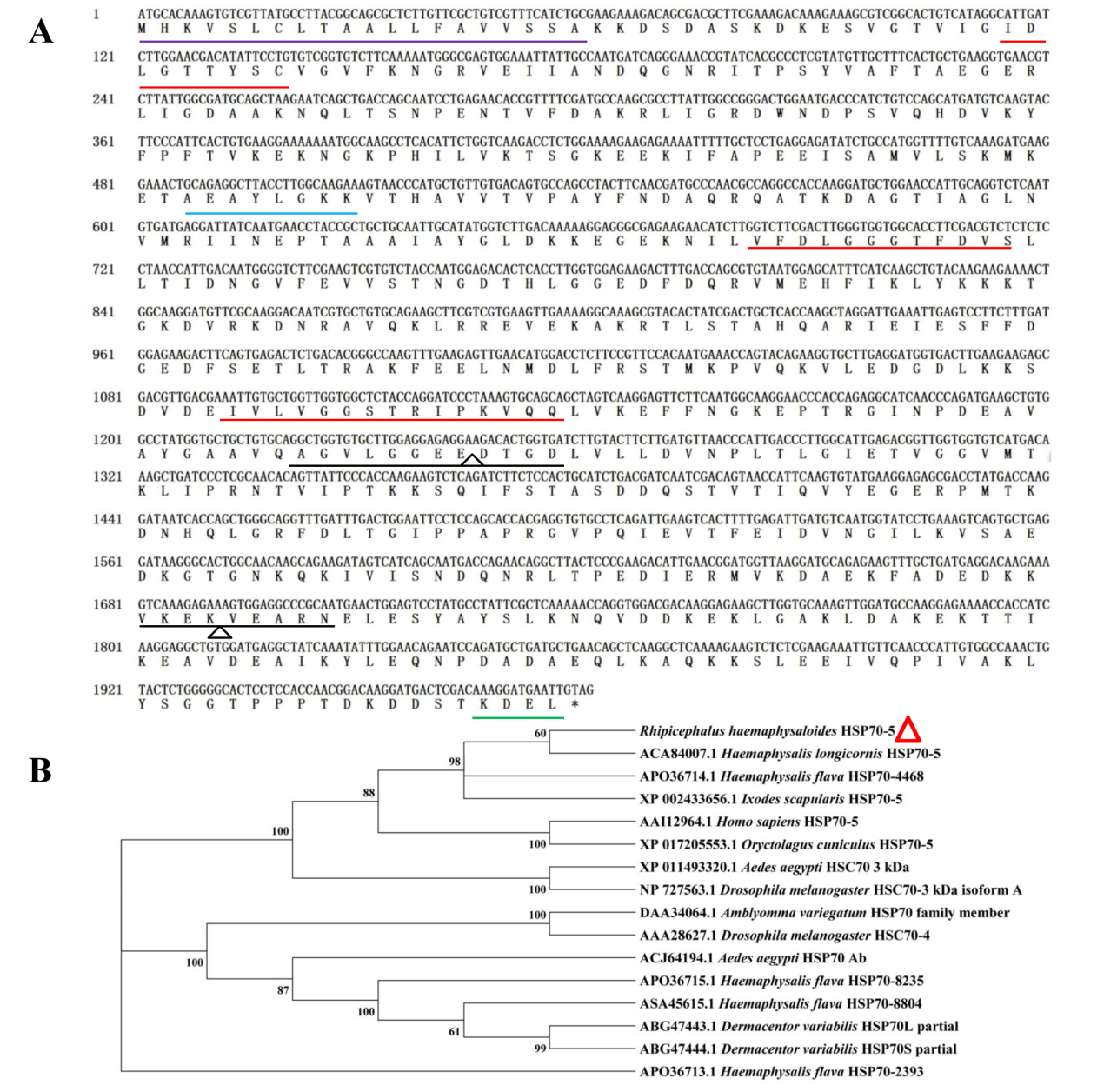
**

**Supplementary Figure 7. Structural analysis of HSP70 gene from the tick *R. haemaphysaloides*. (**A) Nucleotide and predicted amino acid sequence of the cDNA encoding HSP70 gene (GenBank accession. MK584638); (B) Phylogenetic tree HSP70 in ticks and other species was constructed by software MEGA 5.0 with Neighbor-joining tree and 100 bootstrap replications based on available amino acid sequences. (B) Phylogenetic tree of HSP70 genes in ticks and other species was constructed by software MEGA 5.0 with Neighbor-joining tree and 100 bootstrap replications based on available amino acid sequences. HSP70 protein included three signature sequences (highlighted by a red underlines), ATP binding site motif (blue underline), ATP binding domain, substrate binding domain (marked with underline) and the extreme C-terminal endoplasmic reticulum targeting sequence (KDEL, highlighted by a green underline). The signal peptide is marked with purple underline.

## Supplementary Tables

**Supplementary Table 2. Primers for RNA interference of differentially expressed protein.**

| Genes | Primers (5'-3') | Amplicon length |
| --- | --- | --- |
| RNAi-HSP70-1F | ***GGATCCTAATACGACTCACTATAGG***AGGAGGGCGAGAAGAACATCT | 506 bp |
| RNAi- HSP70-1R | CTTGCCATTGAAGAACTCCTTGAC |  |
| RNAi- HSP70-2F | AGGAGGGCGAGAAGAACATCT |  |
| RNAi- HSP70-2R | ***GGATCCTAATACGACTCACTATAGG***CTTGCCATTGAAGAACTCCTTGAC |  |
| Luciferase-RNAi-F1 | ***GGATCCTAATACGACTCACTATAGG***GCTTCCATCTTCCAGGGATAC | 573 bp |
| Luciferase-RNAi-R1 | CGTCCACAAACACAACTCCTCC |  |
| Luciferase-RNAi-F2 | GCTTCCATCTTCCAGGGATACG |  |
| Luciferase-RNAi-R2 | ***GGATCCTAATACGACTCACTATAGG***CGTCCACAAACACAACTCCTC |  |

**Supplementary Table 3. Primers for real-time quantitative PCR of differentially expressed proteins.**

| Genes | Primer-F | Primer-R | Amplicon length |
| --- | --- | --- | --- |
| RHVg-1 | CCGAGCGGAAGCCAGTTGT | CAAGAATGCGTGGAGGATGTTG | 138 bp |
| RHVg-2 | CCAAGTGGCACGCAAACGA | GAGGAAAGAGGCGATACGG | 152 bp |
| RHVg-3 | CGTCGTGGCTGAGGATGGT | GCGTTCCCGTGAAGTGGTA | 137 bp |
| RHVg-4 | GACCTCCCCGTCTACAGCTA | GCGGGAGTCTTCTTGGTCTC | 134 bp |
| HSP70 | GACGAAATTGTGCTGGTTGGTG | GCAGCACCATAGGCCACAG | 128 bp |
| ELF1α | CGTCTACAAGATTGGTGGCATT | CTCAGTGGTCAGGTTGGCAG | 108 bp |
