# Supplementary figures and images for: Ovary Proteome Analysis Reveals RH36 Regulates Reproduction via Vitellin Uptake Mediated by HSP70 Protein in Hard Ticks
Source: Front Cell Infect Microbiol. 2020 Mar 10;10:93. doi: 10.3389/fcimb.2020.00093 (PMC7076983; doi:10.3389/fcimb.2020.00093)

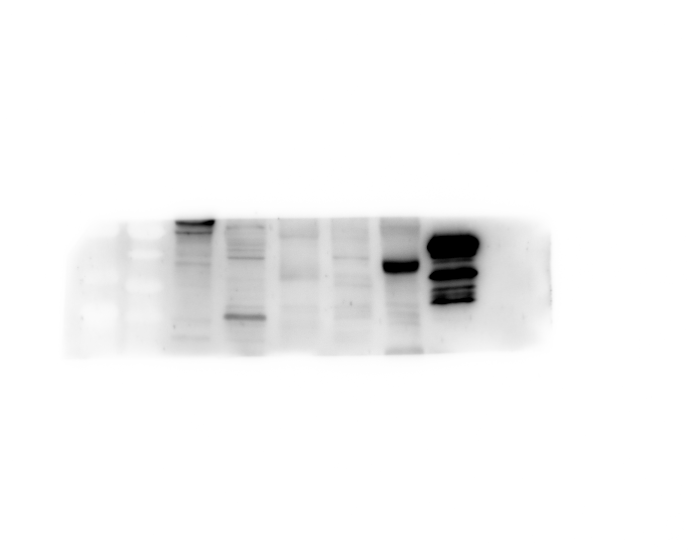

Supplement: Supplementary file 1 [file Data_Sheet_1.zip › Frontiers_Supplementary_Material/original pictures for immunbolotting/Fig. 1/1-RH36 F5.tif]

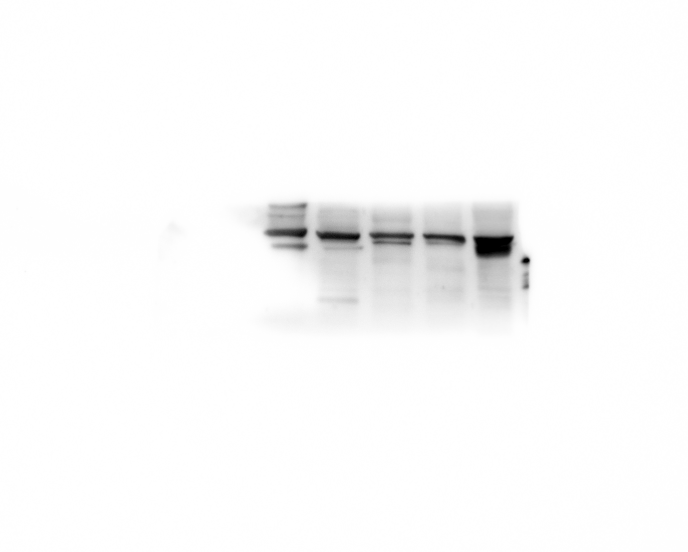

Supplement: Supplementary file 1 [file Data_Sheet_1.zip › Frontiers_Supplementary_Material/original pictures for immunbolotting/Fig. 1/2-actin F5.tif]

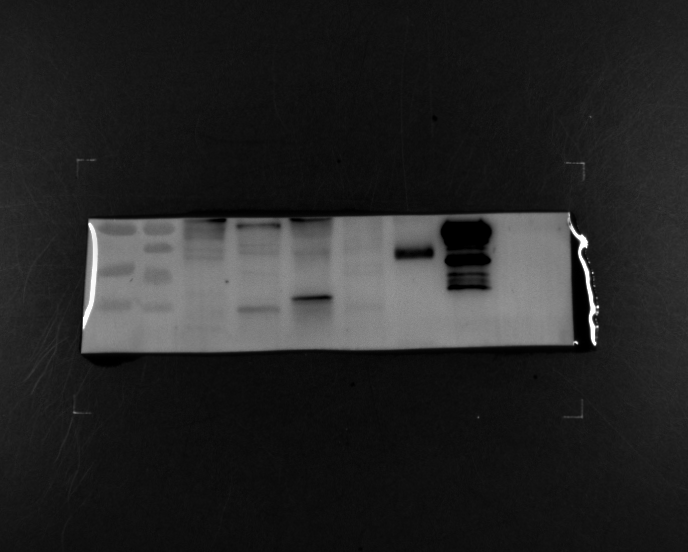

Supplement: Supplementary file 1 [file Data_Sheet_1.zip › Frontiers_Supplementary_Material/original pictures for immunbolotting/Fig. 1/3-RH36 E0.tif]

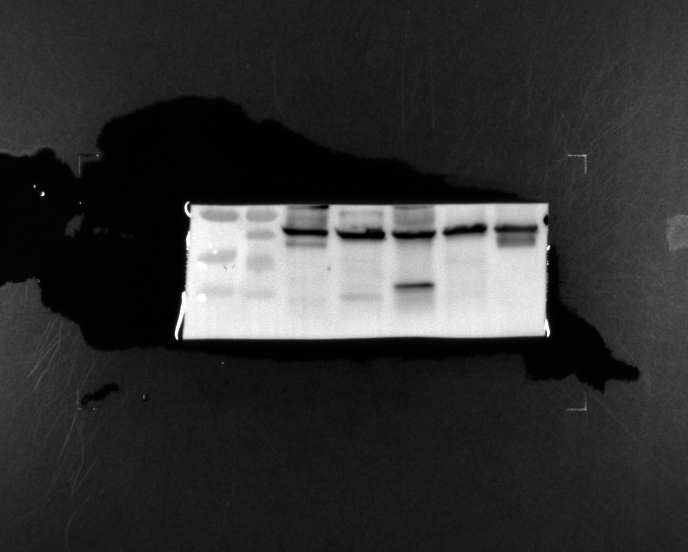

Supplement: Supplementary file 1 [file Data_Sheet_1.zip › Frontiers_Supplementary_Material/original pictures for immunbolotting/Fig. 1/4-actin E0.tif]

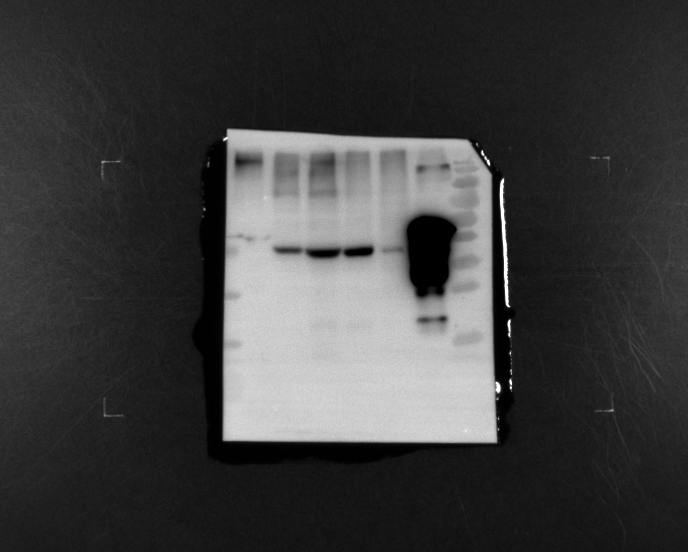

Supplement: Supplementary file 1 [file Data_Sheet_1.zip › Frontiers_Supplementary_Material/original pictures for immunbolotting/Fig. 1/5-RH36 SG.tif]

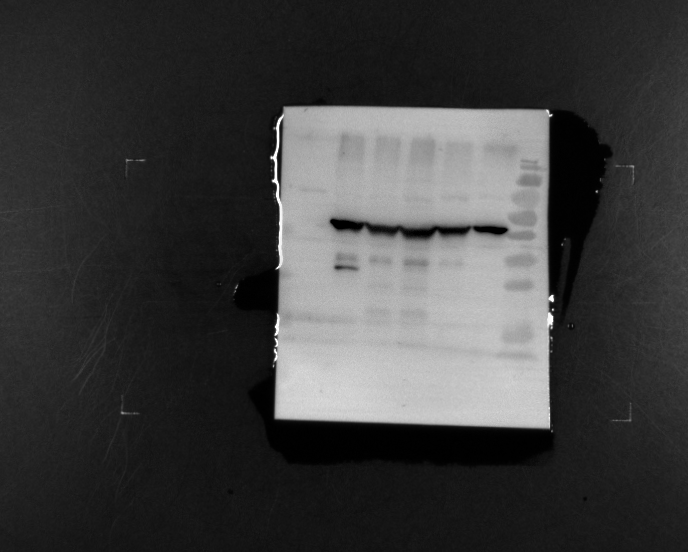

Supplement: Supplementary file 1 [file Data_Sheet_1.zip › Frontiers_Supplementary_Material/original pictures for immunbolotting/Fig. 1/6-actin SG.tif]

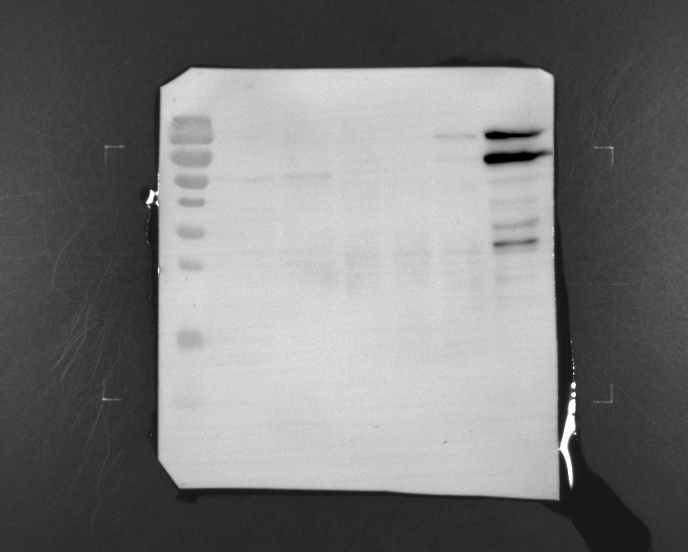

Supplement: Supplementary file 1 [file Data_Sheet_1.zip › Frontiers_Supplementary_Material/original pictures for immunbolotting/Fig. 2/1-Vn FB.tif]

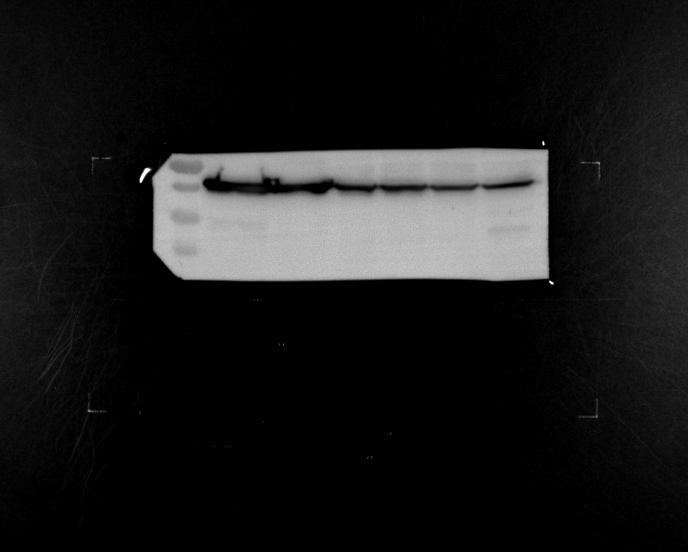

Supplement: Supplementary file 1 [file Data_Sheet_1.zip › Frontiers_Supplementary_Material/original pictures for immunbolotting/Fig. 2/2-Actin FB.tif]

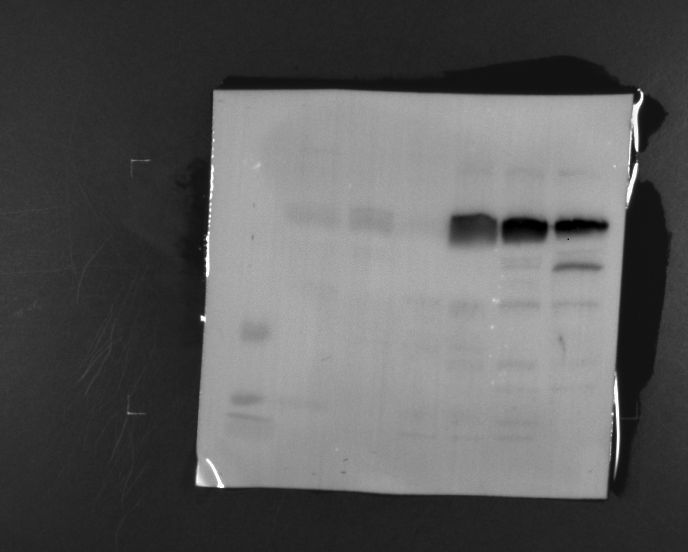

Supplement: Supplementary file 1 [file Data_Sheet_1.zip › Frontiers_Supplementary_Material/original pictures for immunbolotting/Fig. 2/3-Vn HE.tif]

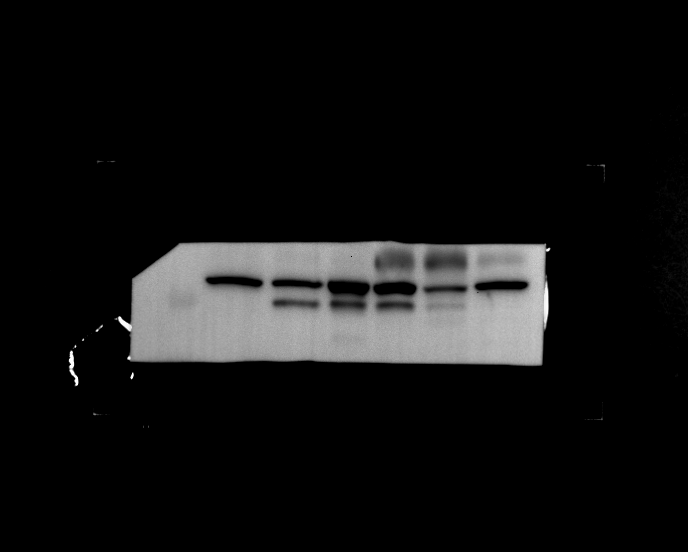

Supplement: Supplementary file 1 [file Data_Sheet_1.zip › Frontiers_Supplementary_Material/original pictures for immunbolotting/Fig. 2/4-Actin HE.tif]

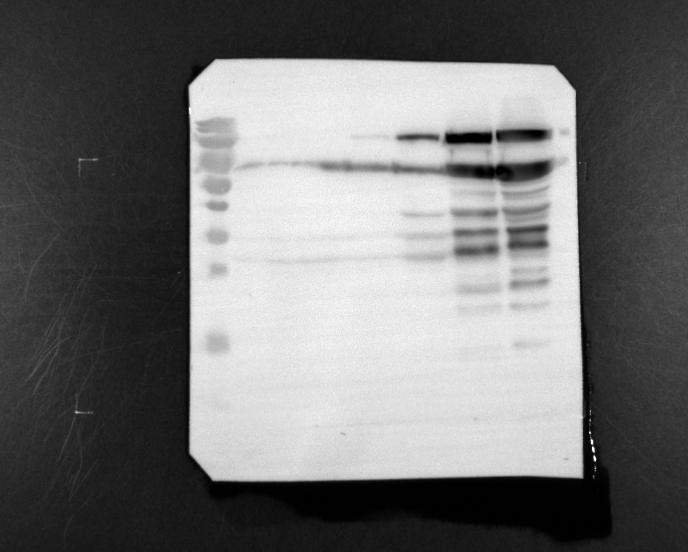

Supplement: Supplementary file 1 [file Data_Sheet_1.zip › Frontiers_Supplementary_Material/original pictures for immunbolotting/Fig. 2/5-Vn OV.tif]

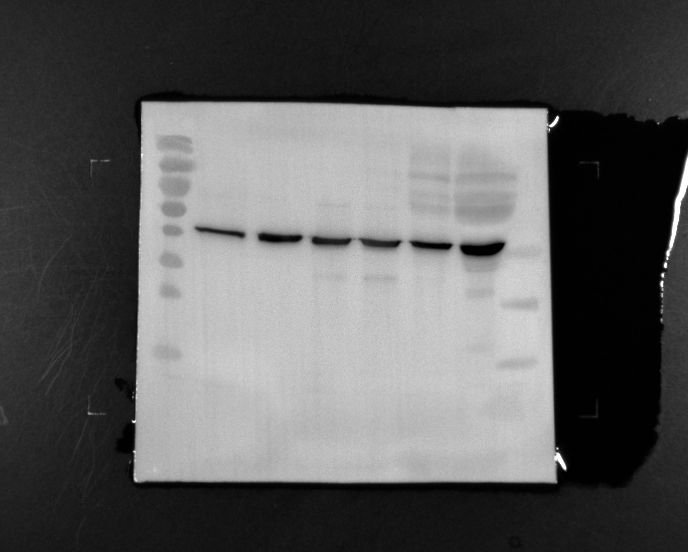

Supplement: Supplementary file 1 [file Data_Sheet_1.zip › Frontiers_Supplementary_Material/original pictures for immunbolotting/Fig. 2/6-Actin OV.tif]

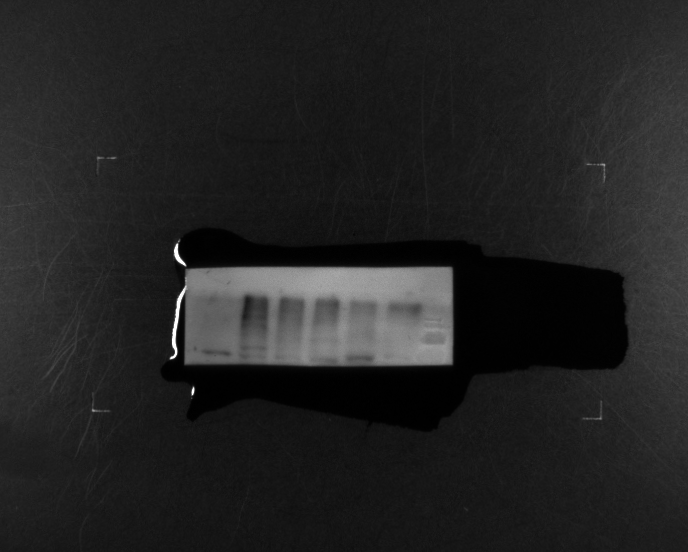

Supplement: Supplementary file 1 [file Data_Sheet_1.zip › Frontiers_Supplementary_Material/original pictures for immunbolotting/Fig. 2/7-Vn SG.tif]

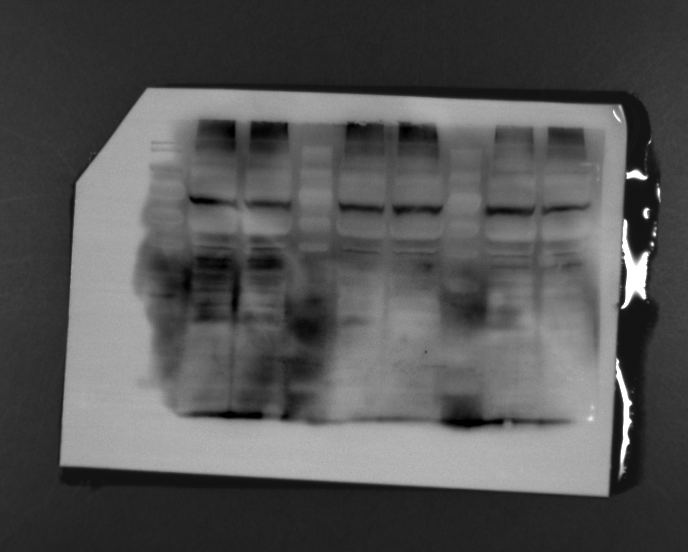

Supplement: Supplementary file 1 [file Data_Sheet_1.zip › Frontiers_Supplementary_Material/original pictures for immunbolotting/Fig. 4/1-Vn F5.tif]

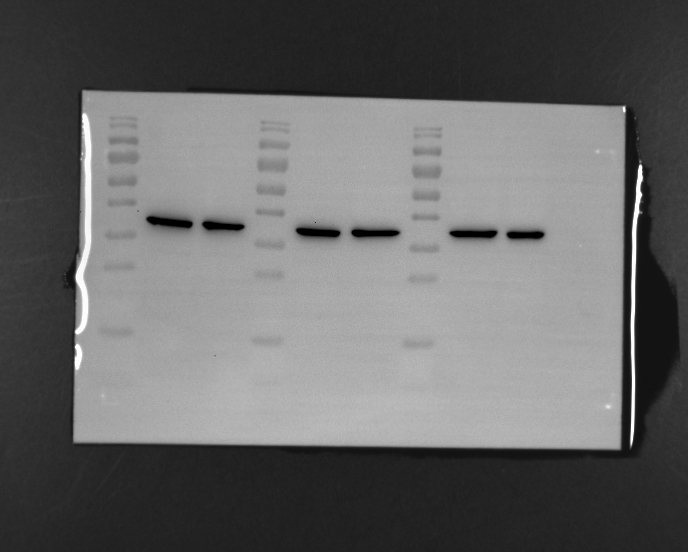

Supplement: Supplementary file 1 [file Data_Sheet_1.zip › Frontiers_Supplementary_Material/original pictures for immunbolotting/Fig. 4/2-GAPDH F5.tif]

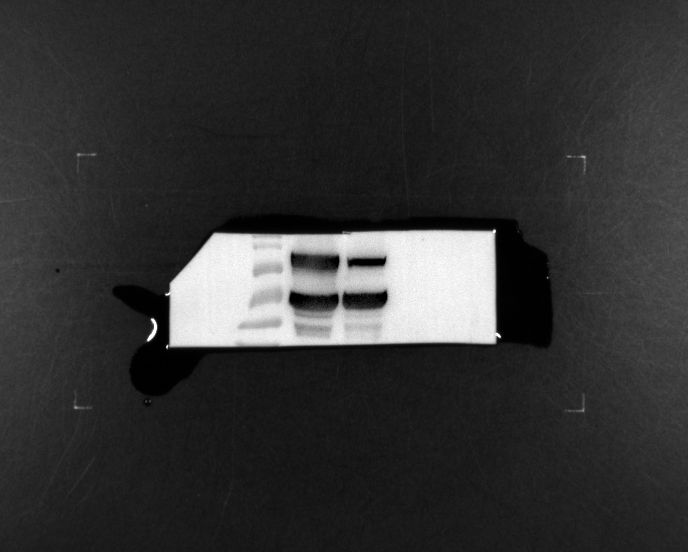

Supplement: Supplementary file 1 [file Data_Sheet_1.zip › Frontiers_Supplementary_Material/original pictures for immunbolotting/Fig. 4/3-Vn E3.tif]

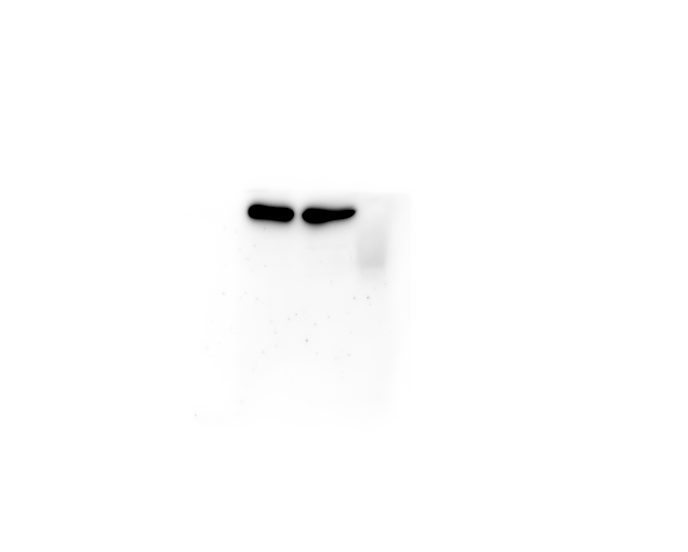

Supplement: Supplementary file 1 [file Data_Sheet_1.zip › Frontiers_Supplementary_Material/original pictures for immunbolotting/Fig. 4/4-GAPDH E3.tif]

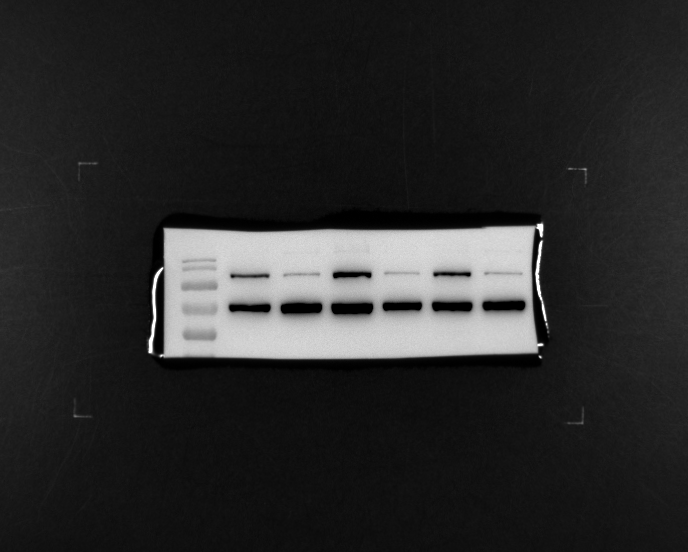

Supplement: Supplementary file 1 [file Data_Sheet_1.zip › Frontiers_Supplementary_Material/original pictures for immunbolotting/Fig. 4/5-Vn E10.tif]

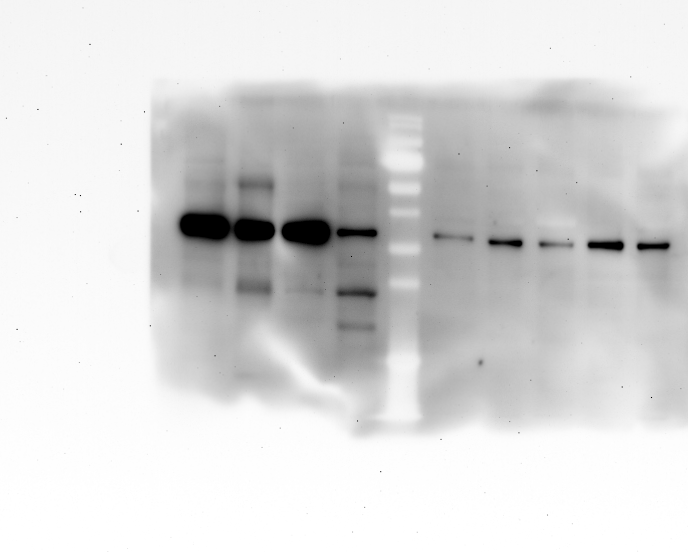

Supplement: Supplementary file 1 [file Data_Sheet_1.zip › Frontiers_Supplementary_Material/original pictures for immunbolotting/Fig. 4/6-GAPDH E10.tif]

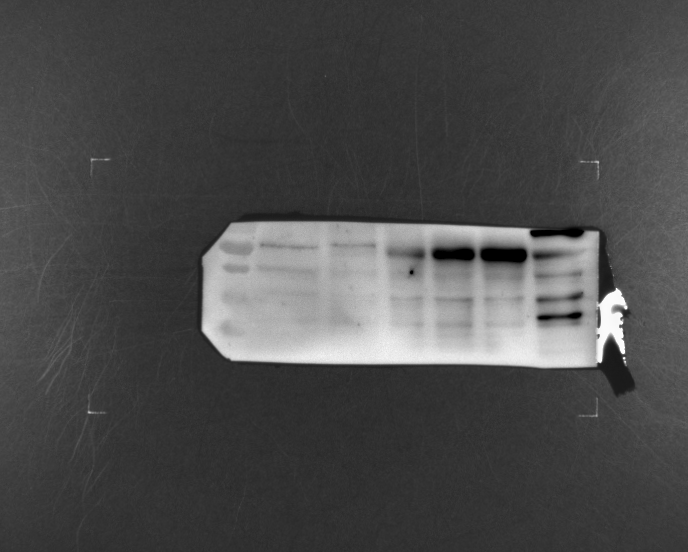

Supplement: Supplementary file 1 [file Data_Sheet_1.zip › Frontiers_Supplementary_Material/original pictures for immunbolotting/Fig. 6/1-HSP70 FB.tif]

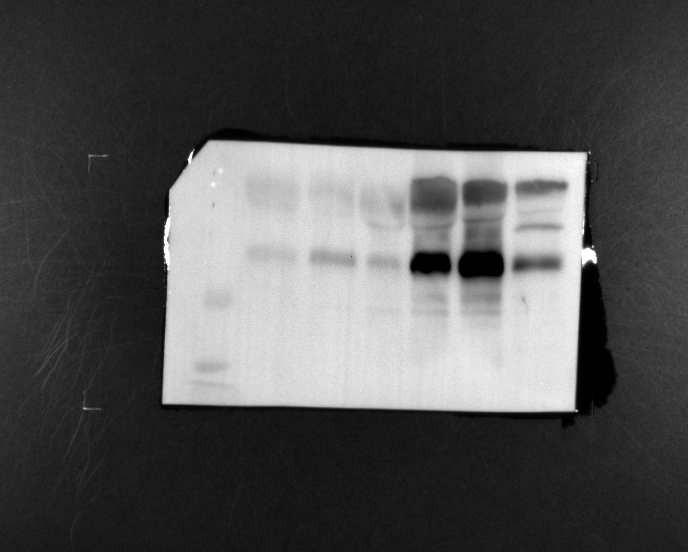

Supplement: Supplementary file 1 [file Data_Sheet_1.zip › Frontiers_Supplementary_Material/original pictures for immunbolotting/Fig. 6/3-HSP70 HE.tif]

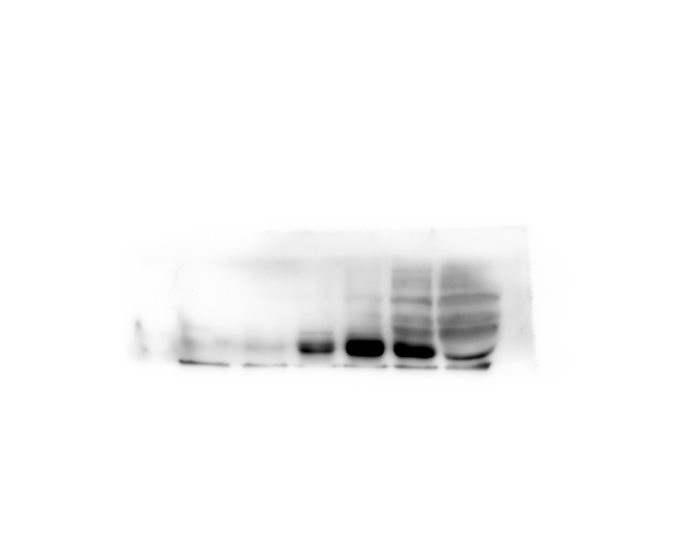

Supplement: Supplementary file 1 [file Data_Sheet_1.zip › Frontiers_Supplementary_Material/original pictures for immunbolotting/Fig. 6/5-HSP70 OV.tif]

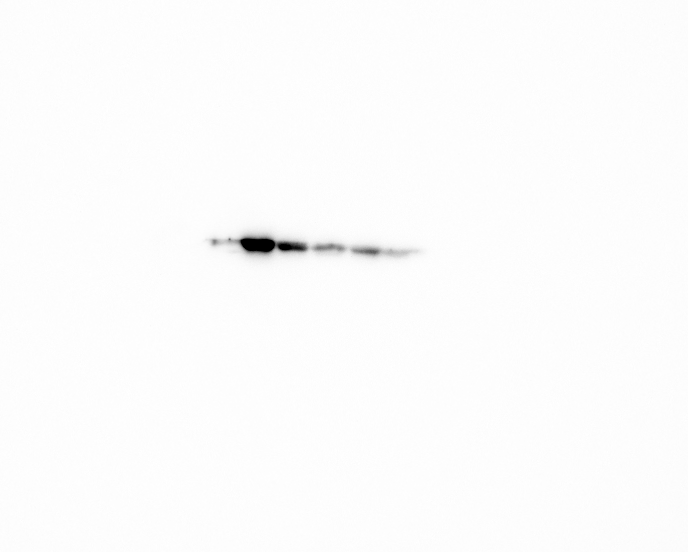

Supplement: Supplementary file 1 [file Data_Sheet_1.zip › Frontiers_Supplementary_Material/original pictures for immunbolotting/Fig. 6/7-HSP70 SG.tif]

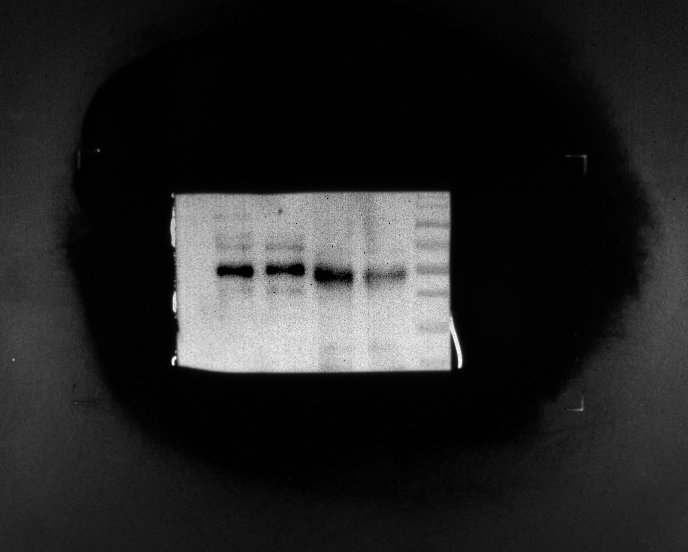

Supplement: Supplementary file 1 [file Data_Sheet_1.zip › Frontiers_Supplementary_Material/original pictures for immunbolotting/Fig. 7/1-HsSP70 F5.tif]

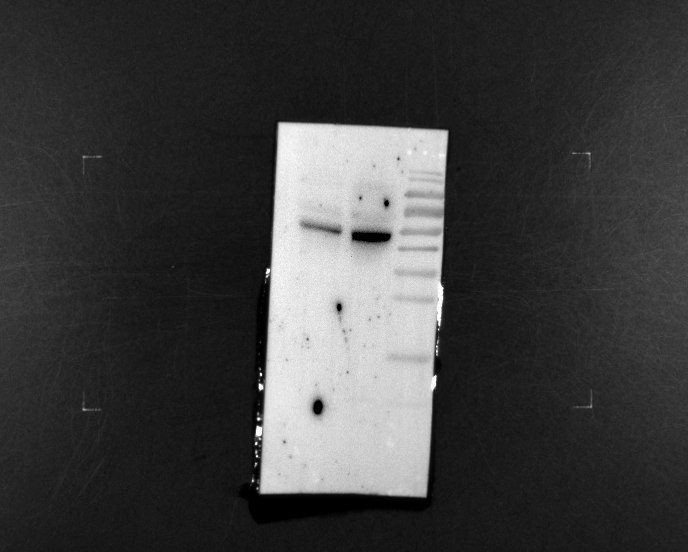

Supplement: Supplementary file 1 [file Data_Sheet_1.zip › Frontiers_Supplementary_Material/original pictures for immunbolotting/Fig. 7/3-HSP70 E3.tif]

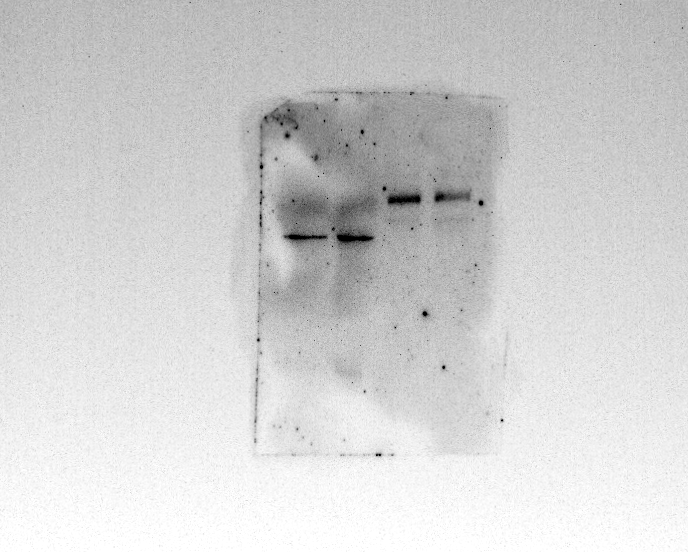

Supplement: Supplementary file 1 [file Data_Sheet_1.zip › Frontiers_Supplementary_Material/original pictures for immunbolotting/Fig. 7/5-HSP70 E10.tif]

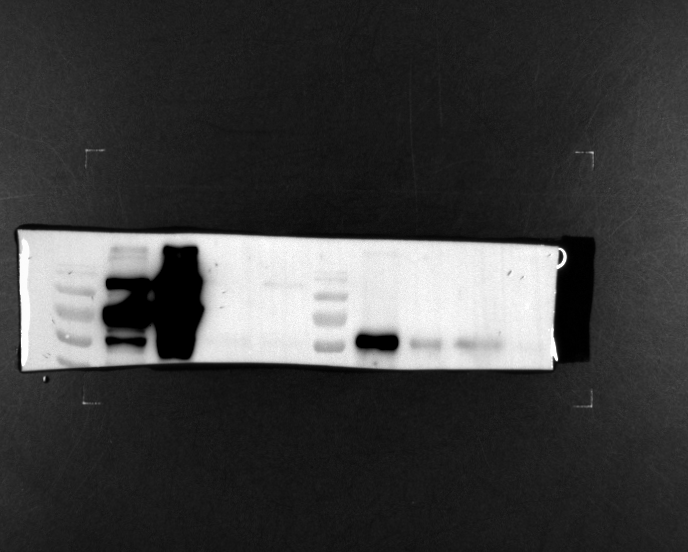

Supplement: Supplementary file 1 [file Data_Sheet_1.zip › Frontiers_Supplementary_Material/original pictures for immunbolotting/Fig. 8/1-HSP70.tif]

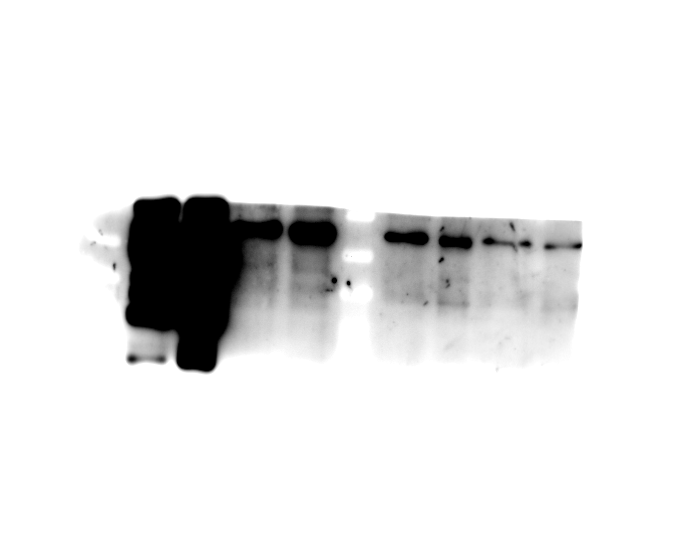

Supplement: Supplementary file 1 [file Data_Sheet_1.zip › Frontiers_Supplementary_Material/original pictures for immunbolotting/Fig. 8/2-GAPDH.tif]

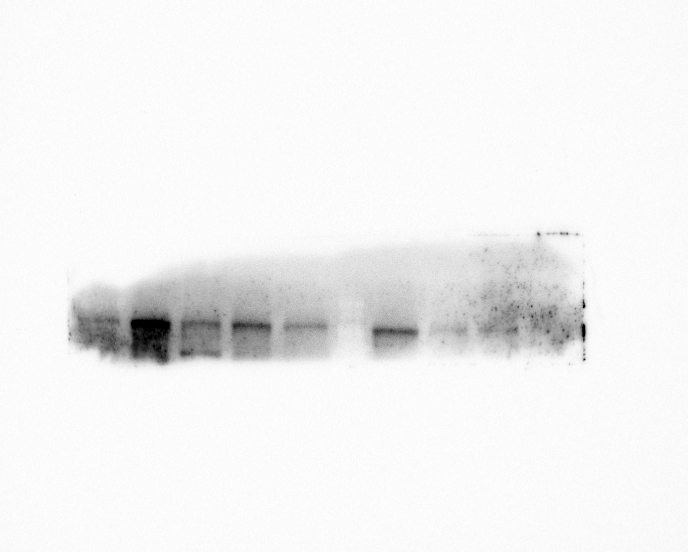

Supplement: Supplementary file 1 [file Data_Sheet_1.zip › Frontiers_Supplementary_Material/original pictures for immunbolotting/Fig. 9/1-Vn.tif]

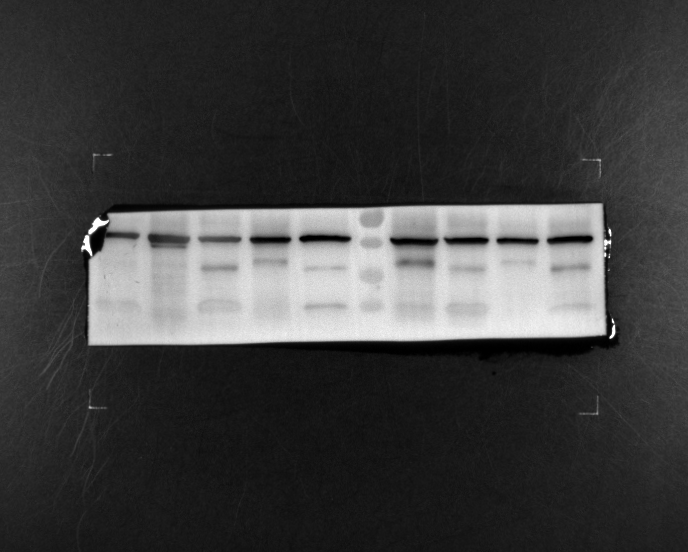

Supplement: Supplementary file 1 [file Data_Sheet_1.zip › Frontiers_Supplementary_Material/original pictures for immunbolotting/Fig. 9/2-actin.tif]
